# Transcriptomic and proteomic analyses of a pale-green durum wheat mutant shows variations in photosystem components and metabolic deficiencies under drought stress

**DOI:** 10.1186/1471-2164-15-125

**Published:** 2014-02-12

**Authors:** Ariadna Peremarti, Caterina Marè, Alessio Aprile, Enrica Roncaglia, Luigi Cattivelli, Dolors Villegas, Conxita Royo

**Affiliations:** 1 Agrotecnio Center, Av. Alcalde Rovira Roure 191, Lleida E-25198, Spain; 2 Field Crops Program, IRTA (Institute for Food and Agricultural Research and Technology), Av. Alcalde Rovira Roure 191, Lleida E-25198, Spain; 3 Consiglio per la ricerca e la sperimentazione in agricoltura – Genomics Research Centre, Via S. Protaso 302, Fiorenzuola d'Arda 29017, (PC), Italy; 4 Department of Environmental and Biological Technologies and Sciences, University of Salento, Provinciale Lecce-Monteroni, Lecce 73100, Italy; 5 Center for Genome Research, Biomedical Sciences Department, Biological Chemistry Section, University of Modena and Reggio Emilia, Via G. Campi 287, Modena 41125, Italy

**Keywords:** Chlorophyll content, Oxidative stress, Pale-green mutant, Proteomics, Transcriptomics, *Triticum turgidum* L var. *durum*, Water stress

## Abstract

**Background:**

Leaf pigment content is an important trait involved in environmental interactions. In order to determine its impact on drought tolerance in wheat, we characterized a pale-green durum wheat mutant (*Triticum turgidum* L. var. *durum*) under contrasting water availability conditions.

**Results:**

The pale-green mutant was investigated by comparing pigment content and gene/protein expression profiles to wild-type plants at anthesis. Under well-watered (control) conditions the mutant had lower levels of chlorophylls and carotenoids, but higher levels of xanthophyll de-epoxidation compared to wild-type. Transcriptomic analysis under control conditions showed that defense genes (encoding e.g. pathogenesis-related proteins, peroxidases and chitinases) were upregulated in the mutant, suggesting the presence of mild oxidative stress that was compensated without altering the net rate of photosynthesis. Transcriptomic analysis under terminal water stress conditions, revealed the modulation of antioxidant enzymes, photosystem components, and enzymes representing carbohydrate metabolism and the tricarboxylic acid cycle, indicating that the mutant was exposed to greater oxidative stress than the wild-type plants, but had a limited capacity to respond. We also compared the two genotypes under irrigated and rain-fed field conditions over three years, finding that the greater oxidative stress and corresponding molecular changes in the pale-green mutant were associated to a yield reduction.

**Conclusions:**

This study provides insight on the effect of pigment content in the molecular response to drought. Identified genes differentially expressed under terminal water stress may be valuable for further studies addressing drought resistance in wheat.

## Background

The leaf chlorophyll content of plants determines their capacity to absorb energy from sunlight and is therefore considered a measure of photosynthetic potential [1]. Plants typically respond to water stress by reducing the leaf chlorophyll content, and hence their photosynthetic activity [2]. Genotypes that tolerate water stress tend to have more leaf chlorophyll and lower canopy temperatures when stressed [1]. In contrast, genotypes that are susceptible to water stress often have higher canopy temperatures [3], and when such plants are well-watered, with their stomata fully open, water evaporation causes leaf cooling [4]. The ability of a genotype to remain green under water stress conditions may therefore enhance photoassimilation, thus increasing productivity and grain filling [5]. In contrast to these general statements, a low level of leaf chlorophyll can limit the interception of light to avoid oxidative damage caused by excess radiation [6] and could be considered a desirable trait under water stress conditions [4]. The optimization of photosynthesis in crops under water stress therefore requires a productive balance between assimilation under favorable conditions and the avoidance of excess radiation under stress [4].

Chlorophylls and carotenoids play an important role in photoassimilation and the synthesis of both molecules is coupled to chloroplast development [7]. Thylakoid organization is coordinated with the expression of nuclear genes, encoding pigment-binding proteins that are imported into the chloroplast to assemble the photosynthetic complexes and the expression of chloroplast encoded genes [8,9]. This process is tightly regulated, so mutants deficient in chlorophyll content can arise not only through the disruption of chlorophyll biosynthesis but also via processes related to photosystem assembly and light-harvesting activity [10,11]. Such chlorophyll mutants have been used to study the function of photosynthetic components as described in rice e.g. [12,13], barley e.g. [14-17] and wheat e.g. [18-20].

Carotenoids are involved in photoprotection and in light harvesting, and different carotenoids play different roles in the photosynthetic complexes. For example, only β-carotene is present in the reaction centers whereas xanthophylls are present in the antennal light harvesting complex (LHC) where each protein has a specific carotenoid complement that ensures maximum photosystem efficiency [21]. Lutein is involved in light harvesting and the quenching of ^3^Chl states [22] and violaxanthin can be replaced by zeaxanthin if the de-epoxidation cycle is activated by photosystem saturation following the absorption of excess light. Zeaxanthin helps to prevent photo-inhibition by dissipating the excess of energy as heat, either by direct quenching or by inducing conformational changes in the LHC proteins [23]. Photo-inhibition is an early consequence of excess light and other environmental stresses, reducing photosynthetic activity and leading to pigment photo-oxidation, the production of reactive oxygen species (ROS) and ultimately cell death [24]. Chloroplasts and mitochondria are the primary sources of ROS, which damage cells by oxidizing nucleic acids, proteins and lipids. However, low concentrations of ROS act as potent signaling molecules that coordinate responses to abiotic stress, pests and pathogens [25]. The balance between ROS production and scavenging by antioxidants can be disrupted by different forms of stress [26].

Wheat is a staple crop that plays a major role in global food security. Conventional breeding strategies have been used to increase grain yields and stress tolerance, but in order to feed a growing population it will be essential to include molecular breeding as well as conventional approaches to achieve further substantial gains [27,28]. Gene expression profiling with microarrays has been used to characterize the response of wheat to heat and drought stress [29], cold [30], *Fusarium* infection [31] and phase-transition [32], and to investigate the molecular basis of grain quality traits [33]. In the context of drought stress, Krugman et al. [34] compared resistant and susceptible wild emmer genotypes to investigate the overlapping regulatory and signaling processes overrepresented in the drought-resistant genotype and to identify candidate genes for drought tolerance. Proteomics has also been used to investigate the response of wheat to nitrogen [35], salinity stress [36,37], low temperatures [38] and drought [39,40]. Poor correlation between transcriptomic and proteomic data has been reported in some stress experiments although such complementary information highlights the multilevel regulation of responses to complex environments [41,42].

Most of the experiments discussed above involved genotypes with contrasting phenotypes and different genetic backgrounds, an approach that allows many genes and stress tolerance strategies to be identified, but makes it difficult to characterize specific genotype-phenotype relationships. This can be achieved in experiments that involve genotypes differing at a single locus e.g. [30]. In order to control for genetic background effects, we compared a pale-green mutant (M) and its wild-type mother variety (WT) to determine the impact of leaf pigment content on gene and protein expression. We studied the chlorophyll and carotenoid content as well as the transcriptomic and proteomic profiles of the mutant and wild-type plants under well-irrigated (control) conditions and terminal water stress. We also tested the performance of the mutant and wild-type plants under irrigated (control) and rain-fed field conditions in order to assess the effect of the mutation on biomass accumulation and grain yield.

## Results

### Chloroplast ultrastructure

**Figure 1:**
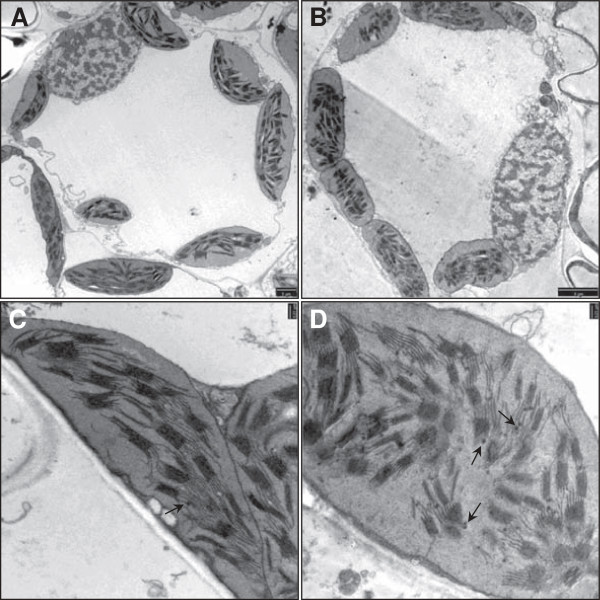
**Chloroplast ultrastructure.** Transmission electron microscopy (TEM) images of a mesophyll cell from **(A)** wild-type and **(B)** pale-green mutant leaf (Bar: 5 μm). **(C)** Detail of wild-type and **(D)** mutant chloroplasts, the latter showing a disorganized pattern of unconnected grana and several plastoglobules (arrows; Bar: 0.23 μm).

Wild-type and mutant leaf sections at the tillering stage were compared by transmission electron microscopy (TEM). As shown in Figure 1, lens-shaped chloroplasts were present in the wild-type mesophyll cells whereas those in the mutant plants were typically cylindrical. The thylakoid grana of the wild-type chloroplasts appeared to be connected by continuous intergranal lamellae parallel to the long axis of the chloroplast, whereas the mutant grana appeared to be disconnected and disorganized within the chloroplast stroma. Osmiophilic lipid globules (plastoglobules) were more abundant in the mutant chloroplasts (shown by arrows in Figure 1C and Figure 1D).

### Physiological responses and field measures

Water stress was applied to plants in the glasshouse by reducing the soil water content at anthesis. This increased the negative value of the midday flag leaf water potential (Ψ_W_), reduced the leaf conductance to water vapor values (g_s_) and reduced the mean net photosynthetic rate (An) but differences between genotypes were not statistically significant (Table 1). In the field, differences in aboveground biomass were significant only under rain-fed conditions, but the grain yield of the mutant plants was reduced by 10.3% under irrigated conditions and by 14.3% under rain-fed conditions, compared to wild-type plants in the same environment (Table 2).

**Table 1:** Analysis of variance and mean values for physiological traits under glasshouse conditions

	**Booting**				**Anthesis**			
	**SWC**	**Ψ**_**W**_	**g**_**s**_	**An**	**SWC**	**Ψ**_**W**_	**g**_**s**_	**An**
	**(%)**	**(Mpa)**	**(mol m**^**-2 **^**s**^**-1**^**)**	**(μmol m**^**-2 **^**s**^**-1**^**)**	**(%)**	**(Mpa)**	**(mol m**^**-2 **^**s**^**-1**^**)**	**(μmol m**^**-2 **^**s**^**-1**^**)**
**A) ANOVA F-values**								
Effect								
Water treatment	0.36 n.s.	0.33 n.s.	0.040 n.s.	1.28 n.s.	405 ^***^	218.5 ^***^	90.1 ^***^	23.5 ^***^
Genotype	0.02 n.s.	0.33 n.s.	0.001 n.s.	1.07 n.s.	1.96 n.s.	3.56 n.s.	0.080 n.s.	0.83 n.s.
Water treatment x genotype	0.05 n.s.	8.22 n.s.	0.100 n.s.	0.97 n.s.	0.52 n.s.	0.61 n.s.	0.001 n.s.	2.00 n.s.
**B) Mean values**								
Water treatment								
Control	47.1	-0.500	0.255	10.03	47.2 ^a^	-0.555 ^a^	0.368 ^a^	15.94 ^a^
Water stress	48.9	-0.525	0.255	10.49	13.5 ^b^	-1.205 ^b^	0.135 ^b^	10.85 ^b^
Control								
Wild-type	46.5	-0.550	0.254	10.32	45.4	-0.580	0.365	14.72
Mutant	47.6	-0.450	0.257	9.73	48.9	-0.530	0.371	17.16
Water stress								
Wild-type	49.0	-0.450	0.259	10.50	12.9	-1.250	0.131	11.11
Mutant	48.7	-0.600	0.251	10.49	14.0	-1.160	0.139	10.58

A) F-values of the analysis of variance and, B) means of soil water content (SWC), flag leaf water potential (Ψ_W_), stomatal conductance (g_s_) and net photosynthetic rate (An) for each water treatment and for the wild-type and the mutant plants under control and water stress conditions. Water stress started at booting. ***P < 0.001, n.s. *P* > 0.05. For each variable, water treatment means with different letters are significantly different for a Tukey test.

**Table 2:** Yield and biomass under field conditions

**Water regime**	**Genotype**	**Yield (kg/ha)**	**Biomass (g/m**^**2**^**)**
Irrigated	WT	7913^a^	1093^a^
	Mutant	7101^b^	1056^a^
Rainfed	WT	5881^a^	859^a^
	Mutant	5041^b^	744^b^

Average values of grain yield and aboveground biomass were determined at anthesis in the wild-type (WT) and the pale-green mutant. Data represent means of 3 years of field experiments. Genotype means with different letters within a water regime differ at *P* = 0.05.

### Pigment content

The total carotenoid and chlorophyll levels in the flag leaves at anthesis under well-watered (control) conditions were significantly lower in the mutant than the wild-type, but differences in antheraxanthin and zeaxanthin levels were not statistically significant (Table 3). Under water stress conditions, only the antheraxanthin content differed significantly between wild-type and mutant leaves, whereas the total carotenoid and chlorophyll contents were similar in both genotypes. The xanthophyll de-epoxidation cycle appeared to be induced in the mutant plants under control conditions. Whereas under terminal water stress, wild-type plants increased the de-epoxidation rate, mutant plants did not. Therefore, there was no statistically significant difference between the genotypes under water stress conditions (Table 3).

**Table 3:** Pigment content in leaves

**Pigment**	**Control**				**Water stress**			
	**WT**	**M**	**M *vs *** **WT (%)**	***P***-**value**	**WT**	**M**	**M *vs *** **WT (%)**	***P***-**value**
Chlorophyll *a*	16308	9704	-40	0.0315	7607	6804	-11	n.s.
Chlorophyll *b*	4700	2374	-49	0.0106	1901	1609	-15	n.s.
Total chlorophylls	21007	12078	-43	0.0247	9508	8413	-12	n.s.
Ratio *a*/*b*	3.46	4.08	18	0.0001	4.03	4.24	5	n.s.
Violaxanthin (V)	968	614	-37	0.0293	535	453	-15	n.s.
Neoxanthin	296	171	-42	0.0095	136	97	-28	n.s.
Antheraxanthin (A)	147	90	-38	n.s.	86	39	-54	0.0152
Lutein	1745	856	-51	0.0071	834	649	-22	n.s.
Zeaxanthin (Z)	253	258	2	n.s.	224	214	-5	n.s.
β-Carotene	1205	734	-39	0.0464	598	566	-5	n.s.
Total carotenoids	4614	2723	-41	0.0162	2357	2019	-14	n.s.
(0.5A + Z)/(VAZ)	0.24	0.32	33	0.0096	0.34	0.33	-3	n.s.

Pigment quantification (μg/g dw) of the flag leaf of wild-type (WT) and pale-green mutant (M) plants at anthesis under control and water stress conditions was performed by HPLC. Values are means of four samples. Xanthophylls de-epoxidation rate [(0.5A + Z)/(VAZ)].

### Transcriptomic profiling

### Microarray quality analysis

**Figure 2:**
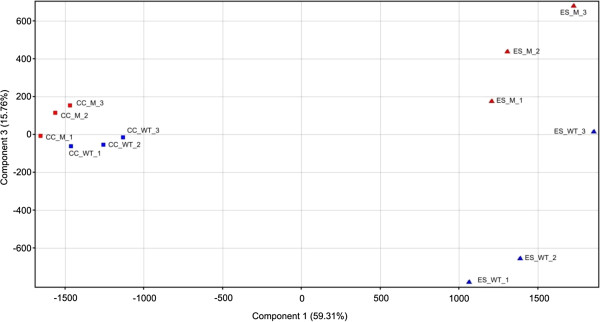
**Principal component analysis plot of the array hybridization data.** The x and y axis represent the two principal components of the total variance 59.31% and 15.76% respectively. The squares indicate the plants grown under control condition (CC), and the triangles indicate the plants grown under water stress (ES). The three biological replicates of each genotype: the pale-green mutant (M) and the wild-type (WT) are shown in the graph.

Microarray data were collected using the Affymetrix Wheat Genome Array and normalized using the RMA algorithm. The average background was 5.33, well within the recommended levels. The percentage of "Present" calls ranged from 39.05% to 42.10% among the 61 k probe sets. The quality of the biological replicates was determined by calculating R^2^ values among replicates of the same sample and the values ranged between 0.988 and 0.997 with an average of 0.995. Principal component analysis highlighted the main sources of total variance. The two components explaining 59.3% and 15.8% of the variance represented the differences between the treatments (control *vs*. water stress) and between the genotypes (pale-green mutant *vs*. wild-type) as shown in Figure 2.

The mutation effect on gene expression was studied comparing the expression of the mutant to wild-type for each water treatment and indicated along the manuscript with the terms induced or repressed genes. In addition, the stress effect was evaluated comparing the gene expression of the water stress to control conditions for each genotype and indicated along the manuscript as up/downregulated genes.

### Gene expression under control conditions

Transcriptional profiling at anthesis revealed differences in gene expression between the two genotypes under control conditions. We identified 23 induced and 15 repressed probe sets in the mutant (>2-fold difference; *P* < 0.05). The array data for selected probe sets were validated by quantitative real-time RT-PCR, showing strong agreement between the fold-change values observed with each method (*r* = 0.96, *P* < 0.0001; Additional file 1: Figure S1).

MIPS FunCatDB was used to distribute the genes into functional categories, revealing that the induced probe sets in the mutant represented chitin catabolism, defense (particularly systemic acquired resistance) and cell wall metabolism (Table 4). The most strongly induced probe sets represented peroxidase 10 (POX10), pathogenesis-related (PR) proteins including beta-glucanases and chitinases, and a TaWRKY45-like transcription factor (Table 5). The repressed probe sets represented functions including systemic interaction with the environment, abscisic acid (ABA) responses, hormonal regulation, stress responses and DNA degradation (Table 4), e.g. sequences representing RAB (responsive to ABA) proteins, late embryogenesis abundant protein LEA1 and endonucleases (Table 5). Probe sets related to cell rescue, defense and virulence (functional category 32) were induced or repressed in the mutant when compared with the wild-type. However, none the subcategories was statistically significant at *P* ≤ 0.005.

**Table 4:** Significant functional categories under control conditions

**Regulation**	**Functional categories**	**Number of genes**	***P***-**value**
Induced	01.05C-compound and carbohydrate metabolism	4	3.81E-03
	01.05.03 polysaccharide metabolism	3	1.21E-04
	01.05.03.03 chitin metabolism	2	1.48E-05
	01.05.03.03.07 chitin catabolism	2	1.48E-05
	32 CELL RESCUE, DEFENSE AND VIRULENCE	4	2.25E-03
	36 SYSTEMIC INTERACTION WITH THE ENVIRONMENT	3	3.46E-03
	36.20 plant/fungal specific systemic sensing and response	3	2.69E-03
	36.20.16 plant defense response	3	1.73E-05
	36.20.16.03 jasmonic acid/ethylene dependent systemic resistance	2	4.88E-05
	36.20.16.05 systemic acquired resistance	2	9.10E-05
Repressed	01.03.16.03 DNA degradation	1	3.37E-03
	32. CELL RESCUE, DEFENSE AND VIRULENCE	4	2.25E-03
	32.01 stress response	4	2.99E-04
	34. INTERACTION WITH THE ENVIRONMENT	4	3.86E-03
	34.11 cellular sensing and response to external stimulus	4	2.65E-03
	34.11.03 chemoperception and response	4	3.12E-04
	34.11.03.12 water response	3	1.53E-05
	36. SYSTEMIC INTERACTION WITH THE ENVIRONMENT	5	8.98E-06
	36.20 plant/fungal specific systemic sensing and response	4	1.48E-04
	36.20.18 plant hormonal regulation	4	7.93E-05
	36.20.18.05 abscisic acid response	3	3.78E-05

Significant functional categories represented in the induced and repressed probe sets in the mutant compared to wild-type plants at anthesis under control conditions according to the MIPS Functional Catalogue Database [122]. Only functional categories with a cut-off of *P* ≤ 0.005 were considered.

**Table 5:** **Modulated probe sets in the mutant compared to the wild**-**type at anthesis under control conditions**

**Expression**	**Probe set**	**Gene annotation**	**Species**^**a**^	**UniProt**	**E-value**	**Fold change**	**qRT-PCR fold expression**
						**(M *vs *** **WT)**	
Induced	TaAffx.128418.43.S1_at	Chitinase 3	*1*	Q8W427	1E-46	5.8	5
	Ta.28.1.S1_at	Beta-1,3-glucanase	*1*	Q4JH28	1E-133	4.9	-
	TaAffx.26815.1.S1_at	Blufensin1	*2*	B8X450	1E-13	4.4	3.4
	Ta.169.1.S1_x_at	Germin-like protein (GLP2b)	*1*	Q9SM34	1E-154	4.3	2.8
	Ta.20188.1.S1_a_at	Unknown				4	-
	TaAffx.6092.1.S1_at	Naringenin,2-oxoglutarate 3-dioxygenase	*3*	B6SZR4	1E-126	3.6	3.5
	Ta.3647.1.S1_at	Putative uncharacterized protein Sb01g018690	*4*	C5WXG4	6E-51	3.2	-
	TaAffx.98744.1.S1_at	Putative uncharacterized protein Sb05g022350	*4*	C5Y4X4	3E-60	3.2	-
	Ta.15159.1.S1_at	Pathogenesis-related protein precursor	*2*	P93181	3E-05	3.1	-
	Ta.20188.1.S1_x_at	Unknown				3.1	-
	TaAffx.24475.1.S1_x_at	Glucan endo-1,3-beta-glucosidase GII	*2*	P15737	9E-19	3.1	-
	Ta.22572.1.S1_at	Unknown				2.7	-
	Ta.8614.2.S1_x_at	WRKY45-like transcription factor	*1*	F8WPI8	2E-53	2.7	2.2
	Ta.3830.1.S1_a_at	Unknown				2.6	-
	Ta.30028.1.S1_s_at	Peroxidase 10 (POX10)	5	Q5I3E8	1E-66	2.4	
	Ta.3830.2.S1_x_at	Unknown				2.3	-
	TaAffx.119315.2.S1_x_at	Beta-glucanase	*2*	Q7M1K2	5E-72	2.2	-
	Ta.2278.3.S1_x_at	Chitinase II (CHT2)	*1*	Q9XEN3	3E-74	2.2	-
	Ta.3830.3.S1_x_at	Unknown				2.1	-
	TaAffx.24475.1.S1_at	Glucan endo-1,3-beta-glucosidase GII	*2*	P15737	9E-19	2.1	-
	Ta.16980.1.S1_at	Peroxidase 10 (POX10)	*5*	Q5I3E8	5E-10	2.1	-
	Ta.16599.1.S1_x_at	Adhesive/proline-rich protein	*3*	B4FH66	8E-29	2.1	-
	Ta.8228.1.S1_at	Agmatine coumaroyl transferase-1(ACT-1)	*2*	A9ZPJ6	0	2	2.5
Repressed	Ta.2638.1.S1_at	RAB protein	*1*	Q41579	1E-175	-3.9	0.08
	Ta.13255.1.S1_at	Dehydrin (WZY1-1)	*1*	Q8W192	6E-93	-3.9	0.3
	Ta.23797.1.S1_x_at	Late embryogenesis abundant protein (LEA1)	*1*	Q8GV49	1E-104	-3.6	-
	Ta.24453.1.S1_s_at	Oxalate oxidase (Germin protein)	*1*	Q9LD27	7E-39	-3.3	-
	TaAffx.98394.1.S1_at	Q-type C2H2 zinc finger protein (ZFP23)	*1*	B0ZYY9	1E-97	-3.3	0.7
	Ta.10390.1.S1_at	Papain-like cysteine proteinase (PAP-14)	*2*	B4ESF2	0	-2.9	-
	Ta.5497.1.A1_at	Endonuclease	*2*	O81958	3E-13	-2.8	-
	Ta.6015.1.S1_at	TdS40 protein (Fragment), putative	*6*	C4WYH7	5E-83	-2.7	-
	TaAffx.83636.1.S1_at	Unknown				-2.7	-
	Ta.4110.1.S1_at	Indole-3-acetic acid-amido synthetase (GH3.2), probable	*7*	P0C0M2	1E-124	-2.4	-
	Ta.5879.1.S1_at	Peptide chain release factor subunit 1 (ERF1), putative	*7*	Q75K79	2E-92	-2.4	-
	Ta.13682.1.A1_at	Progesterone 5-beta-reductase, putative	*7*	Q60DC5	1E-35	-2.3	-
	Ta.7543.1.S1_at	Unknown				-2.2	-
	Ta.5497.1.A1_x_at	Endonuclease	*2*	O81958	3E-13	-2.1	-
	Ta.12477.1.S1_at	Homeobox-leucine zipper protein (ATHB-6)	*3*	B6U539	3E-03	-2.1	-

^a^Species: 1: *Triticum aestivum, 2: Hordeum vulgare, 3: Zea mays, 4: Sorghum vulgare, 5: Triticum monococcum, 6: Triticum turgidum* ssp. *Durum, 7: Oryza sativa.*

-: not determined.

### Gene expression under water stress

**Figure 3:**
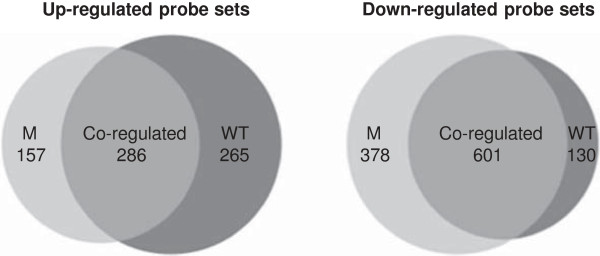
**Modulated probe-sets.** Venn diagrams show the number of modulated probe sets by the wild-type (WT) and mutant (M) at anthesis under water stress conditions compared with the control.

We initially compared each genotype under stress and control conditions to identify both general and genotype-specific stress-modulated genes, which were defined as upregulated (induced by water stress) or downregulated (repressed by water stress), as mentioned above. We identified a total of 1817 probe sets that were modulated by water stress in at least one of the genotypes (Figure 3), including 1282 genes in the wild-type plants, 1422 genes in the mutant plants and 887 in both.

We also compared the wild-type and mutant plants under water stress conditions, to identify genes that were differently-expressed under stress. We identified 166 probe sets that were defined either as induced or repressed in the mutant plants, according to the level of expression under water stress conditions.

#### Genotype-independent stress-response genes

From the 887 probe sets modulated in both genotypes, 286 were upregulated and 601 were downregulated under water stress (Figure 3). The upregulated genes predominantly represented the following functional categories: stress response, carbon compound and carbohydrate metabolism, metabolite transport, amino acid metabolism, ABA response and proline biosynthesis (Additional file 2: Table S1). Genes already known to be associated with water stress are shown in the Additional file 3: Table S2. These include genes encoding sucrose synthase (SUS3, SUS4), glucose-6-phosphate dehydrogenase (G6PD4), branched-chain-amino-acid aminotransferase (BCAT3) and branched-chain alpha-keto acid dehydrogenase E1 α subunit (BCKDHE1α), delta 1-pyrroline-5-carboxylate synthetase 2 (P5CS2), pyrroline-5-carboxylate reductase (P5CR) and RAB18. The downregulated genes predominantly represented the following functional categories: phosphate and secondary metabolism, e.g. 1-aminocyclopropane-1-carboxylate synthase (ACS) and 12-oxophytodienoate reductase (OPR); interaction with the environment, e.g. chitinases, β-glucanases and pathogenesis-related proteins; immune response, e.g. transcription factor WRKY; and phenylpropanoid metabolism, e.g. phenylalanine ammonia-lyase. These genotype-independent stress-modulated genes correspond to the well-characterized drought stress response of wheat, confirming that the mutant plants preserve much of the stress-response signaling network that allows wheat plants to tolerate water stress [34,43].

#### Genotype-dependent stress-response genes in wild-type plants

We identified 395 probe sets that were modulated solely in the wild-type plants or to a greater extent (above the 2-fold threshold) than in the mutant plants. This included 265 upregulated and 130 downregulated genes (Figure 3). The upregulated genes predominantly represented carbohydrate and pyruvate metabolism and the glyoxylate cycle, whereas the downregulated represented metabolism, interaction with the environment, protein modification and signaling pathways (Table 6). Selected genes specifically modulated by the wild-type plants under stress are listed in Table 7, including carbon fixation enzymes such as malate dehydrogenase (NADP-ME3) and phosphoenolpyruvate caboxykinase (PCK1), and glyoxylate cycle enzymes such as isocitrate lyase (ICL) and malate synthase (MLS).

**Table 6:** **Significant functional categories in the wild**-**type under water stress**

**Regulation**	**Functional categories**	**Number of genes**	***P***-**value**
Upregulated	01 METABOLISM	63	6.57E-11
	01.01.11 metabolism of the pyruvate family (alanine, isoleucine, leucine, valine) and D-alanine	3	1.90E-03
	01.01.11.04 metabolism of leucine	3	3.37E-04
	01.01.11.04.02 degradation of leucine	3	1.49E-05
	01.03.16.03 DNA degradation	2	8.84E-04
	01.05C-compound and carbohydrate metabolism	33	2.11E-10
	01.05.02 sugar, glucoside, polyol and carboxylate metabolism	21	1.96E-08
	01.05.03 polysaccharide metabolism	9	9.71E-06
	01.05.05C-1 compound metabolism	5	4.35E-03
	01.05.07.04C-3 compound anabolism	2	3.23E-03
	02 ENERGY	8	4.60E-03
	02.04 glyoxylate cycle	4	2.08E-07
	36.25 animal specific systemic sensing and response	4	3.33E-03
	36.25.16 immune response	4	2.61E-03
	42.01 cell wall	6	4.09E-03
	70.10.03 chromosome	7	1.71E-05
Downregulated	01 METABOLISM	33	3.37E-09
	01.04 phosphate metabolism	17	1.29E-06
	01.05C-compound and carbohydrate metabolism	11	1.73E-03
	01.20 secondary metabolism	5	3.56E-03
	01.20.35 metabolism of secondary products derived from L-phenylalanine and L-tyrosine	3	3.70E-03
	01.20.35.01 metabolism of phenylpropanoids	3	2.81E-03
	14 PROTEIN FATE (folding, modification, destination)	16	2.28E-03
	14.07 protein modification	14	5.23E-04
	14.07.03 modification by phosphorylation, dephosphorylation, autophosphorylation	12	3.51E-05
	20.01.01.07 anion transport	3	1.90E-03
	30.01.05 enzyme mediated signal transduction	6	6.96E-04
	30.01.05.01 protein kinase	6	5.69E-05
	30.01.05.01.06 serine/threonine kinase	5	8.08E-05
	30.05 transmembrane signal transduction	5	1.14E-03
	30.05.01 receptor enzyme mediated signalling	5	6.10E-04
	30.05.01.12 transmembrane receptor protein tyrosine kinase signalling pathways	5	1.54E-05
	32 CELL RESCUE, DEFENSE AND VIRULENCE	10	2.00E-03
	32.01 stress response	9	1.59E-04
	32.01.06 cold shock response	4	6.69E-04
	34 INTERACTION WITH THE ENVIRONMENT	11	1.77E-03
	34.11 cellular sensing and response to external stimulus	11	7.70E-04
	34.11.03.12 water response	3	2.94E-03
	34.11.09 temperature perception and response	5	4.24E-04
	70.03 cytoplasm	8	3.86E-03

Significant functional categories represented in the specifically upregulated and downregulated probe sets in wild-type plants at anthesis under water stress conditions compared to well-watered (control) conditions according to the MIPS Functional Catalogue Database [122]. Only functional categories with a cut-off of *P* ≤ 0.005 were considered.

**Table 7:** Selected genotype-dependent regulated genes under water stress

**Probe set ID**	**Gene name**	**Arabidopsis ID**	**Log**_**2 **_**ratio WT stress/WT control**	**Log**_**2 **_**ratio M stress/M control**	**Log**_**2 **_**ratio M/WT under water stres**
**PHOTOSYNTHESIS AND PIGMENT SYNTHESIS**					
Ta.23025.1.A1_at	LHB1B2; Light-harvesting complex II chlorophyll a/b binding protein 1	AT2G34420	1.14	-	-
Ta.28496.1.A1_x_at			-	2.04	1.02
Ta.30702.1.S1_x_at			-	1.63	-
Ta.3249.2.S1_x_at			-	1.13	-
Ta.3249.3.A1_at			-	1.20	-
Ta.18362.1.A1_at	ELIP1; Early light-inducible protein	AT3G22840	-1.80	-	1.41
Ta.23419.1.S1_x_at			-1.37	-	0.95
Ta.8718.2.S1_at	ATPD; F-type H^+^ -transporting ATPase subunit delta	AT4G09650	-	1.47	-
Ta.8718.2.S1_x_at			-	1.29	-
Ta.3243.1.S1_at	HEMA1; Glutamyl-tRNAreductase 1	AT1G58290	-	1.52	-
Ta.3243.1.S1_x_at			-	1.47	-
Ta.20830.1.A1_at	RUBISCO small subunit; Ribulose bisphosphate carboxylase small chains. chloroplast precursor	TC371960^b^	-	1.36	1.04
TaAffx.128414.24.A1_s_at	RUBISCO large subunit	ATCG00490	-	-1.64	-0.85
Ta.17243.1.S1_at	MVA1; Acetyl-CoA C-acetyltransferase/hydroxymethylglutaryl-CoA synthase	AT4G11820	-	1.13	-
Ta.3366.1.S1_at	DXS; 1-deoxy-D-xylulose-5-phosphate synthase; CLA1 (Cloroplastos Alterados 1);	AT4G15560	-	1.55	-
Ta.20776.1.S1_at	PSY; Phytoene synthase	AT5G17230	-	1.07	-
**OXIDATIVE STRESS AND ROS SIGNALING**					
**Detoxification**					
Ta.11386.2.S1_a_at	TAPX; Thylakoidal ascorbate peroxidase	AT1G77490	-	1.25	-
Ta.11386.2.S1_x_at			-	1.31	-
Ta.488.2.S1_at	APX4; Ascorbate peroxidase 4	AT4G09010	-	1.7	1.73
Ta.23079.1.S1_a_at	APX1; Ascorbate peroxidase 1	AT1G07890	-	-1.03	-
Ta.30028.1.S1_s_at	PER12; Peroxidase 12	AT1G71695	-	-1.62	-
TaAffx.613.2.A1_x_at	Peroxidase. putative	AT4G31760	1.12	-	-
Ta.18560.1.S1_at		AT4G33420	-	-1.78	-
Ta.21505.1.S1_at		AT1G49570	-	-1.29	-
Ta.30697.1.S1_at		AT5G05340	-	-1.05	-
TaAffx.39568.2.S1_at		AT5G58390	-	-1.57	-
Ta.6127.1.S1_at	Peroxiredoxin type 2. putative	AT3G52960	1.22	-	-
TaAffx.100459.1.S1_at	Glutaredoxin family protein	AT3G62950	1.32	-	-
Ta.8571.1.S1_x_at	Glyoxalase I family protein	AT1G80160	-	-1.03	-1.07
TaAffx.79142.1.S1_at	ATGLX1; Glyoxalase I homolog; lactoylglutathione lyase	AT1G11840	-	-0.76	-
Ta.8571.1.S1_a_at	Glyoxalase I family protein; lactoylglutathione lyase family protein	AT1G80160	-	-1.04	-
Ta.8571.1.S1_x_at			-	-1.03	-1.07
Ta.23704.1.S1_s_at	ATGSTF13; Glutathione transferase	AT3G62760	-	-1.36	-
Ta.303.2.S1_at			-	-1.08	-
Ta.3681.1.S1_x_at	ATGSTU19; Glutathione S-transferase TAU 19	AT1G78380	-1.02	-	-
TaAffx.79276.1.S1_x_at	ERD9; Early-responsive to dehydration 9; glutathione transferase	AT1G10370	-	-1.23	-
Ta.21001.1.S1_at	GGT1; Gamma-glutamyl transpeptidase 1	AT4G39640	-	-0.99	-
TaAffx.64682.1.S1_at	RBOHD; Respiratory burst oxidase homologue D	AT5G47910	-	1.07	-
**Signaling**					
Ta.4936.1.S1_at	CIPK2; CBL-Interacting protein kinase 2	AT5G07070	1.23	-	-
Ta.8465.1.S1_at	CIPK6; CBL-Interacting protein kinase 6	AT4G30960	1.46	-	-
Ta.5041.1.S1_at	CIPK23; CBL-Interacting protein kinase 23	AT1G30270	-	-1.59	-1.06
Ta.5041.2.S1_a_at			-	-1.22	-
Ta.5272.3.S1_x_at	CBL1; Calcineurin B-like protein 1	AT4G17615	-	-1.04	-
Ta.236.1.S1_at	MAPK3; MAP kinase 3	AT3G45640	-	-1.20	-
**CARBOHYDRATE METABOLISM**					
**Galactose and Raffinose family metabolism**					
Ta.26092.1.S1_at	SIP1; Putative galactinol--sucrose galactosyl transferase 1; Raffinose synthase	AT1G55740	1.06	-	-
Ta.6023.1.S1_at	SIP2; Putative galactinol--sucrose galactosyl transferase 2	AT3G57520	1.20	-	-
Ta.7541.2.S1_a_at	GalAK; Galactokinase	AT3G10700	1.27	-	-
**Trehalose synthesis**					
Ta.20649.1.S1_x_at	ATTPS6; Alpha,alpha-trehalose-phosphate synthase 6	AT1G68020	1.47	-	-1.14
**Starch degradation**					
Ta.10281.1.S1_a_at	AMY1; Alpha-amylase-like	AT4G25000	1.21	-	-
Ta.16135.1.A1_at	CT-BMY; Chloroplast Beta-Amylase	AT4G17090	-1.51	-	-
TaAffx.68872.1.S1_at			-1.25	-	-
Ta.8827.1.S1_at	DPE2; Disproportionating enzyme 2	AT2G40840	-	1.09	-
**Sucrose hydrolisis**					
Ta.9000.1.S1_at	BFRUCT3; Beta-fructosidase; invertase, vacuolar	AT1G62660	1.51	-	-1.33
Ta.2788.1.A1_at	Sucrose:sucrose 1-fructosyltransferase	B5TK35^a^	-	1.48	-
Ta.2789.2.S1_at	Sucrose:fructan 6-fructosyltransferase	Q96466^a^	-	1.49	1.18
**Cellulose synthesis**					
Ta.12382.1.S1_at	ATCSLB02; Cellulose synthase	AT2G32620	1.04	-	-
Ta.12382.1.S1_x_at			1.11	-	-
Ta.7192.1.S1_at	ATCSLC12; Cellulose synthase like C12	AT4G07960	1.15	-	-
Ta.4447.1.S1_at	CESA1; Cellulose synthase 1	AT4G32410	1.24	-	-
Ta.4447.1.S1_x_at			1.40	-	-
Ta.4447.2.S1_x_at	CESA3; Cellulose synthase 3	AT5G05170	1.16	-	-
Ta.12382.3.S1_at	ATCSLB05; Cellulose synthase	AT4G15290	-	1.09	-
Ta.5628.1.A1_at	IRX9; Irregular xylem 9; xylosyltransferase	AT2G37090	1.81	-	-1.30
Ta.29111.1.A1_at	XT2; UDP-Xylosyltransferase 2	AT4G02500	1.26	-	-1.32
Ta.13337.1.S1_at	Xyloglucan:xyloglucosyl transferase, putative	AT5G57540	1.38	-	-1.59
Ta.13337.2.S1_at			1.21	-	-1.41
Ta.13337.2.S1_x_at			1.44	-	-1.57
**TCA AND RELATED PATHWAYS**					
**Pyruvate metabolism and glyoxylate cycle**					
Ta.18775.1.S1_at	NADP-ME4; NADP-malic enzyme 4	AT1G79750	1.11	-	-
Ta.25543.2.S1_at	NADP-ME3; Malate dehydrogenase (oxaloacetate-decarboxylating)	AT5G25880	1.20	-	-
Ta.13280.1.S1_a_at	PCK1; Phosphoenolpyruvate carboxykinase	AT4G37870	1.05	-	-
Ta.13280.2.A1_at			1.10	-	-
Ta.23970.1.A1_x_at	MLS; Malate synthase	AT5G03860	1.92	-	-1.31
Ta.23989.1.A1_at	ICL; Isocitrate lyase	AT3G21720	2.38	-	-1.90
TaAffx.79218.1.S1_at			1.63	-	-1.35
Ta.19423.1.S1_a_at	PDC2; Pyruvate decarboxylase-2	AT5G54960	-	-1.14	-
Ta.13281.1.S1_at	Pyruvate decarboxylase. putative	AT5G01320	-	-1.07	-
**Amino acid metabolism**					
Ta.15150.1.S1_at	MCCA; Methylcrotonoyl-CoA carboxylase subunit alpha	AT1G03090	1.09	-	-
Ta.15150.1.S1_x_at			1.12	-	-
Ta.30569.1.S1_a_at	MCCB; Methylcrotonoyl-CoA carboxylase beta chain	AT4G34030	1.10	-	-
Ta.12252.1.S1_s_at	BCE2; 2-oxoisovalerate dehydrogenase E2 component (dihydrolipoyltransacylase)	AT3G06850	1.13	-	-
**Glutamate metabolism**					
TaAffx.129066.1.S1_at	GAD; Glutamate decarboxylase 1	AT5G17330	-	-1.08	-
Ta.25990.1.A1_at	GAD3; Glutamate decarboxylase 3	AT2G02000	-	-1.42	-1.05
Ta.25990.1.A1_x_at			-	-1.53	-1.11
Ta.1870.1.S1_a_at	GDH2; Glutamate dehydrogenase 2	AT5G07440	-	-1.31	-1.23
Ta.21001.1.S1_at	GGT1; Gamma-glutamyl transpeptidase 1	AT4G39640	-	-0.99	-
Ta.30684.1.S1_at	AO; L-aspartate oxidase	AT5G14760	-	-1.06	-
**REGULATORS OF CARBON METABOLISM AND SENSING**					
Ta.12733.1.S1_at	SAL1; Inositol or phosphatidylinositol phosphatase	AT5G63980	-	-1.02	-
Ta.4492.1.S1_at	PI3P5K; Phosphatidylinositol-3-phosphate 5-kinase	AT1G71010	-	1.02	-
Ta.10130.1.S1_at	RAP2.12; Related to AP2 12	AT1G53910	1.05	-	-1.32
Ta.13336.1.S1_at	RAP2.7; Related to AP2 7	AT2G28550	1.68	-	-1.05
Ta.21035.1.S1_at	RAP2.8; RAV2 (Regulator of the ATPase of the vacuolar membrane)	AT1G68840	-	-2.02	-1.44
Ta.27316.1.S1_at			-	-1.11	-
TaAffx.18447.1.S1_at	BZIP63; Basic Leucine Zipper 63; BZO2H3	AT5G28770	-	-1.84	-1.05
TaAffx.18447.3.S1_s_at			-	-2.16	-1.42
TaAffx.18447.5.S1_s_at			-	-1.55	-1.03
**CHLOROPLAST TRANSCRIPTION AND TRANSLATION**					
TaAffx.27156.1.S1_at	RNA polymerase beta' subunit-2	ATCG00170	-	1.24	-
TaAffx.107547.1.S1_at	RNA polymerase beta' subunit-1	ATCG00180	-	1.08	-
TaAffx.113923.1.S1_at	Chloroplast DNA-dependent RNA polymerase B subunit	ATCG00190	-	1.26	-
Ta.769.1.S1_at	PTAC16; Plastid transcriptionally active 16	AT3G46780	-	1.08	-
TaAffx.128946.4.S1_at	Chloroplast ribosomal protein S2	ATCG00160	-	1.18	-
**OTHERS**					
Ta.5161.3.S1_at	CRK29; Cystein-rich RLK (Receptor-like protein kinase) 29	AT4G21410	-	-1.73	-
Ta.5161.3.S1_x_at			-	-1.32	-
Ta.6870.2.S1_a_at	MKK6; MAP kinase kinase 6	AT5G56580	-	-1.08	

Selected genes were specifically significant regulated by the wild-type (WT) and the mutant (M) under water stress compared with the same genotype under well-watered (control) conditions. Last column (M vs WT) compares mutant versus WT expression under water stress. Positive and negative values indicate upregulation and downregulation respectively, whereas no statistically significant differences are indicated with a dash (-).

^a^UniProt ID from *Triticum turgidum* subsp. *durum*.

^b^Blast executed against TaGI (DFCI) Wheat Gene Index: TC371960. homologue to UniRef100_P26667.

#### Genotype-dependent stress-response genes in mutant plants

We identified 535 probe sets that were modulated solely in the mutant plants or to a greater extent (above the 2-fold threshold) than in the wild-type plants. This included 157 upregulated and 378 downregulated genes representing diverse functional categories (Figure 3; Table 8). The most significant categories in the upregulated group were associated with chloroplast transcription, interactions with the environment (cellular sensing, response to external stimuli, chemoperception and response), stress responses and metabolism, whereas the downregulated group was dominated by functional categories such as interaction with the environment (response to external stimuli), stress responses and plant defense (Table 8). The downregulated genes also included those induced in the mutant under control conditions.

**Table 8:** Significant functional categories in the mutant under water stress

**Regulation**	**Functional categories**	**Number of genes**	***P***-**value**
Upregulated	01 METABOLISM	26	3.94E-03
	11.02.03.01 general transcription activities	3	8.94E-04
	11.02.03.01.04 transcription elongation	3	3.51E-06
	32 CELL RESCUE. DEFENSE AND VIRULENCE	12	1.61E-03
	32.01 stress response	11	5.80E-05
	32.01.03 osmotic and salt stress response	4	3.98E-03
	34 INTERACTION WITH THE ENVIRONMENT	16	4.91E-05
	34.11 cellular sensing and response to external stimulus	14	2.11E-04
	34.11.03 chemoperception and response	8	4.89E-03
	34.11.03.13 osmosensing and response	4	4.19E-03
	36.20.18 plant hormonal regulation	7	2.53E-03
	36.20.18.02 ethylen response	3	3.86E-03
Downregulated	01 METABOLISM	72	1.44E-09
	01.04 phosphate metabolism	28	2.37E-04
	01.05C-compound and carbohydrate metabolism	24	1.03E-03
	01.05.03.03 chitin metabolism	3	1.33E-04
	01.05.03.03.07 chitin catabolism	3	1.33E-04
	01.20 secondary metabolism	11	3.27E-04
	01.20.35 metabolism of secondary products derived from L-phenylalanine and L-tyrosine	5	2.65E-03
	01.20.35.01 metabolism of phenylpropanoids	5	1.73E-03
	01.20.35.01.03 metabolism of lignins	4	3.58E-04
	14.07.03 modification by phosphorylation. dephosphorylation. autophosphorylation	20	8.53E-04
	16.13C-compound binding	4	2.49E-03
	16.17.01 calcium binding	7	2.97E-04
	16.25 oxygen binding	8	3.57E-04
	20 CELLULAR TRANSPORT. TRANSPORT FACILITIES AND TRANSPORT ROUTES	37	1.41E-05
	20.01 transported compounds (substrates)	29	1.30E-04
	32 CELL RESCUE. DEFENSE AND VIRULENCE	29	6.36E-07
	32.01 stress response	20	3.59E-06
	32.01.01 oxidative stress response	9	1.79E-05
	32.01.06 cold shock response	7	2.23E-04
	32.07 detoxification	8	8.06E-04
	32.07.07 oxygen and radical detoxification	8	7.13E-04
	32.07.07.05 peroxidase reaction	5	6.49E-04
	34 INTERACTION WITH THE ENVIRONMENT	35	1.35E-08
	34.11 cellular sensing and response to external stimulus	34	3.56E-09
	34.11.03 chemoperception and response	16	5.00E-04
	34.11.05 mechanical stimulus perception and response	2	1.86E-03
	34.11.09 temperature perception and response	10	2.62E-05
	34.11.10 response to biotic stimulus	14	4.46E-08
	36 SYSTEMIC INTERACTION WITH THE ENVIRONMENT	19	3.43E-06
	36.20 plant/fungal specific systemic sensing and response	18	3.84E-06
	36.20.16 plant defense response	7	3.72E-05
	36.20.16.05 systemic acquired resistance	3	1.96E-03
	36.20.18 plant hormonal regulation	12	1.42E-03
	40.10 cell death	6	1.02E-04
	40.10.02 apoptosis (type I programmed cell death)	6	9.85E-06
	40.10.02.01 anti-apoptosis	6	7.34E-08
	70.02 eukaryotic plasma membrane/membrane attached	12	4.61E-04

Significant functional categories represented in the specifically upregulated and downregulated probe sets lists in the mutant at anthesis under water stress conditions compared to well-watered (control) conditions according to the MIPS Functional Catalogue Database [122]. Only functional categories with a cut-off of *P* ≤ 0.005 were considered.

The genes that were upregulated specifically in the mutant included those involved in the synthesis of carotenoid precursors, e.g. hydroxymethylglutaryl-CoA synthase (MVA1), 1-deoxy-D-xylulose-5-phosphate synthase (DXS) and phytoene synthase (PSY), and those involved in chloroplast gene expression, photosynthesis, chlorophyll binding and detoxification, e.g. glutamyl-tRNA reductase 1 (HEMA1), light harvesting complex II components such as LHB1B2 and ATPase subunit delta, and the Rubisco small subunit. We observed the downregulation of several genes representing glutamate metabolism such as glutamate decarboxylase (GAD), glutamate dehydrogenase (GDH), γ-glutamyltranspeptidase (GGT) and L-aspartate oxidase (AO); and several signaling pathway components, including calcineurin B-like protein 1 (CBL1), inositol phosphatase (SAL1) and MPK3 (Table 7).

#### Differentially-expressed stress-response genes under stress conditions

We compared the transcriptional profiles of wild-type and mutant plants under water stress and identified 166 differently-expressed probe sets (29 induced and 137 repressed in the mutant). The only functional category significantly represented among the 29 induced probe sets was the channel/pore transport class (Additional file 4: Table S3), corresponding to the transcripts for two plasma membrane intrinsic proteins (PIP2;2, PIP2;5) that were downregulated in both genotypes but to a lesser extent in the mutant (Additional file 3: Table S2). Other genes that were induced in the mutant under water stress included those encoding ascorbate peroxidase (APX4), sucrose: fructan 6-fructosyltransferase (SFT6), *myo*-inositol oxygenase (MIOX1) and dihydrodipicolinate reductase 3 (crr1) (Table 7 and Additional file 3: Table S2).

The genes that were repressed in the mutant plants under stress were predominantly related to carbohydrate metabolism, peroxidase/oxidative stress responses, the glyoxylate cycle and chromosome subcellular localization (Additional file 4: Table S3). Representative repressed genes included those encoding RAP2.8, GDH, GAD, CBL-interacting protein kinase 23 (CIPK23) and glyoxalase (Table 7). We also found genes encoding ICL, MLS and trehalose phosphate-synthase (TPS) which were not affected by stress in mutant plants but were upregulated in wild-type plants, thus appearing in the repressed transcript category (Table 7).

### Proteomic profiling

The effect of the mutation was also investigated by carrying out a comparative proteomic analysis between wild-type and mutant plants at anthesis under the same conditions as described above.

When wild-type and mutant plants were compared under well-watered (control) conditions, we matched 182 protein spots (data not shown) among which two showed a significant (>2-fold) difference in signal intensity between genotypes, although neither spot could be identified because of the low abundance of the corresponding protein. Even so, this experiment supported the transcriptomic and suggested that there were only minor differences between the genotypes under normal conditions.

When we compared each genotype under stress and control conditions, we matched 207 protein spots among both genotypes. From the 207, we found that 32 spots were modulated by water stress in the wild-type plants and 62 were modulated in the mutant plants, including 25 spots that were common to both genotypes. This left seven modulated spots specific to the wild-type plants and 37 specific to the mutant.

When the two genotypes were compared under water stress, we identified 28 spots that were more abundant in the mutant and 17 that were less abundant in the mutant. We selected 19 of the induced spots and all 17 of the repressed spots based on the likelihood of extracting useful amounts of protein, and we identified 13 proteins from the induced set and 8 from the repressed set (Additional file 5: Figure S2). The proteins were identified by peptide mass fingerprinting (Additional file 6: Table S4) and MS/MS analysis (Additional file 7: Table S5). The induced proteins predominantly represented carbohydrate and amino acid metabolism, photosynthesis, detoxification and phytoalexin biosynthesis, whereas the repressed proteins predominantly represented photosynthesis (Table 9). Two proteins that were specifically upregulated in the mutant were the oxygen-evolving enhancer protein 2 corresponding to the PSBP subunit of photosystem II (A.n. Q00434), and catalase-1 (CATA1; A.n. Q00434). Two proteins that were specifically downregulated in the mutant were the Rubisco small subunit chain PW9 (A.n. P26667) and the photosystem I reaction center subunit IV (A.n. P13194).

**Table 9:** Differently-expressed proteins in the mutant (M) compared to the wild-type (WT) under water stress

**Expression**	**Function**	**Protein description**	**SwissProt accession number**	**Species**^**b**^	**Ratio WT stress/WT control**	**Ratio M stress/M control**	**Ratio M/T under water stres**	**Method**^**c**^
Induced	Carbohydrate metabolism	G3PX; Glyceraldehyde-3-phosphate dehydrogenase. cytosolic	P26517	1	-	-	304.7	PMF
		G3PX; Glyceraldehyde-3-phosphate dehydrogenase. cytosolic	P26517	1	-	-	215.4	PMF
		Glyceraldehyde-3-phosphate dehydrogenase A. chloroplastic	P09315	2	-	170.3	87.8	MS/MS
		Fructose-bisphosphatealdolaseF2CR16	BAJ85287^a^	1	-	-	3.7	MS/MS
	Amino acid metabolism	Cysteine synthase	P38076	3	-	-	252.7	MS/MS
		Chloroplast aspartate aminotransferase	ACG59771^a^	3	8.7e^-13^	-	101.1	MS/MS
		Putative 3-beta hydroxysteroid dehydrogenase/isomerase protein	BAJ86066^a^	1	-	134.5	75.8	MS/MS
	Photosynthesis	ATPB; ATP synthase subunit beta. chloroplastic	P20858	3	-	-	114.2	PMF
		PSBO; Oxygen-evolving enhancer protein 1. chloroplastic (PSII)	Q40459	4	-	-	2.5	PMF
		PSBP; Oxygen-evolving enhancer protein 2. chloroplastic (PSII)	Q00434	3	-	2.1	2.1	PMF
	Biosynthesis phytoalexins	Isoflavone reductase	B5M699	1	-	-	2.6	PMF
	Translation	Elongation factor Tu. chloroplastic-like. predicted	XP_003575279^a^	5	-	2.2	2.5	MS/MS
	Detoxification	CATA1; Catalase-1	Q43206	3	-	3.4	2.5	PMF
Repressed	Photosynthesis	Chloroplast light-harvesting chlorophyll a/b binding protein (Lhc II typeI CAB)	ADL41158^a^	3	0.4	0.3	0.5	MS/MS
		Photosystem I reaction center subunit IV. chloroplastic (PSI RCsubIV)	P13194	1	-	0.5	0.4	MS/MS
		Precursor of CP29. core chlorophyll a/b binding (CAB) protein (PSII Lhc4)	CAA44777^a^	1	595.9	265.5	0.4	MS/MS
		RBS2; Ribulose bisphosphate carboxylase small chain PW9. chloroplastic	P26667	3	-	0.5	0.4	PMF
		Chlorophyll a-b binding protein CAB 1B-21 (PSI Lhc I)	ACO06083^a^	3	-	-	0.3	MS/MS
		Chlorophyll a-b binding protein 1B-21. chloroplastic (CAB 1B-21)	Q9SDM1	1	-	2.5e^-13^	0.002	MS/MS
	Heme binding	HBL2; Non-symbiotic hemoglobin 2	O24521	6	-	0.5	0.5	PMF
		C71AJ; Cytochrome P450 71A19	Q9T0K0	6	0.3	0.4	0.4	PMF

^a^Accession number from NCBI.

^b^Species: 1: *Hordeum vulgare*; 2: *Zea mays*; 3:*Triticum aestivum*; 4: *Nicotiana tabacum*; 5: *Brachypodium distachyon*; 6: *Arabidopsis thaliana*.

^c^Method: PMF: Peptide Mass Fingerprinting; MS/MS: MALDI TOF/TOF.

Ratios are only indicated for differences statistically significant.

## Discussion

### Chloroplast ultrastructure and pigment content

Chlorotic mutants with affected chloroplast ultrastructure have been described in several reports [9,14,44-46]. The chloroplasts in these plants often contain fewer grana structures than wild-type plants, and thinner thylakoid membranes that are not stacked in the normal manner [47]. In contrast, the chloroplasts in our pale-green mutant contained abundant grana with normally-stacked thylakoids, but their distribution within the stroma was disorganized, potentially reflecting a lack of membrane continuity or the degradation of inter-grana lamella membranes. The stacking adhesion of the thylakoids in grana structures is regulated by the phosphorylation of photosystem II proteins, which also ensures photosynthetic efficiency [45]. We found no significant difference in the photosynthetic rate of the wild-type and mutant and no quantitative differences in transcripts or proteins representing the photosynthetic apparatus at anthesis under control conditions.

The mutant chloroplasts also contained more stromal plastoglobules than wild-type chloroplasts. These lipoprotein bodies are structurally continuous with the thylakoid membranes and facilitate content exchange with the thylakoid lumen [48]. They may also be involved in thylakoid development and disassembly, increasing in abundance in response to oxidative stress and delivering the antioxidant molecules stored on them to the thylakoid lumen, where they scavenge ROS [49,50]. The structure of the inter-grana lamella in the mutant chloroplasts could therefore reflect thylakoid reorganization or the impact of oxidative stress already at tillering stage under field capacity conditions.

Although the pigment content of the mutant plants was ~41% lower than in wild-type plants under control conditions, the photosynthetic rate was similar (Table 1). This seems illogical given that carotenoids and chlorophylls are required for the proper folding, assembly and stability of LHC apoproteins [51]. However, we detected a greater xanthophyll de-epoxidation rate in the mutant as indicated by the relationship (0.5A + Z)/(VAZ). The zeaxanthin in the mutant may compensate for the lack of other xanthophylls in the antenna proteins [52], and may also preserve the structure of the photosystem and its membranes by dissipating photochemical energy as heat to prevent photo-oxidation [22]. This may explain the abnormal chloroplast ultrastructure in the mutant but the near-normal photosynthetic rate. Our data suggest that, under control conditions, the mutant compensates for the lower pigment content and that minor oxidative stress has no detrimental effect on photosynthetic capacity or biomass accumulation.

However, under water stress conditions the level of de-epoxidation increases in wild-type plants, and both genotypes show similar values for the relationship (0.5A + Z)/(VAZ) (Table 3). This suggests that the mutant has a lower capacity for increasing the de-epoxidation rate. An alternative explanation is that the pool of convertible violaxanthin is rapidly depleted and only non-convertible violaxanthin remains to maintain the structural integrity of the antenna [53]. Because xanthophylls help to prevent photo-oxidation, the mutant plants without additional de-epoxidated carotenoids to cope for the water stress damage may sense higher oxidative stress in the chloroplast under water stress. Thus, the pale-green mutant may be more susceptible since the ability of plants to withstand oxidative stress determines the level of overall stress tolerance [54].

### Differences between the wild-type and mutant transcriptomes at anthesis (no stress)

There were only minor differences in gene expression between the wild-type and mutant plants under control conditions at anthesis, mirroring the similar net photosynthetic rates (Table 1). The few repressed genes in the mutant were predominantly related to ABA responses and the induced genes mostly represented pathogenesis-related (PR) proteins and peroxidases involved in plant defense (Table 5).

Apart from their role in defense, peroxidases may be modulated by cellular redox disturbances [55]. In such cases, nuclear gene expression can be regulated by ROS-dependent signal transduction or low-molecular-weight antioxidants produced in the chloroplast. For example, the expression of PR proteins and peroxidases increases in ascorbate-deficient mutants [56]. Since no lesion or pathogen attack was detected in our investigation, it is most likely that low-level of oxidative stress triggers defense gene expression in the pale-green mutant. Proteins involved in plant defense are also sensitive to hormones such as salicylic acid (SA), jasmonate (JA) and ethylene, but we did not find any differently-expressed genes related to these pathways in the mutant. Plant defense signaling pathways are also activated through WRKY transcription factors that bind to pathogen-response and wound-inducible promoter elements (W-boxes and GCC-like elements). The *TaWRKY45* gene was induced in the mutant, and may help to activate some of the PR genes we also identified. This transcription factor confers resistance to *Fusarium* head blight (FHB) in wheat [57] and increases the expression of PR-1 and PR-2 genes in rice to confer resistance to *Pseudomonas syringae*, salinity and drought tolerance, as well as reducing ABA sensitivity [58].

Genes encoding defense enzymes that synthesize secondary metabolites such as phenylpropanoids and flavonoids were also induced in the mutant, including naringerin, 2-oxoglutarate 3-dioxygenase and agmatine coumaroyltransferase (ACT), the latter representing the final step in the hydroxycinnamic acid amide (HCAA) synthesis pathway. Peroxidases can also couple agmatine conjugates to form antifungal compounds known as hordatines in barley and (to a lesser extent) in wheat [59]. Finally, the blufensin-1 gene was also induced in the mutant. This is a negative regulator conferring sensitivity to powdery mildew in barley by modulating penetration resistance [60].

The repressed probe sets were related to systemic interactions with the environment, especially the ABA response, e.g. genes encoding dehydrins such as RAB proteins and other LEA proteins that accumulate during seed maturation and in vegetative tissues in response to abiotic stress [61]. RAB is a LEA family dehydrin that is induced by ABA, and the Arabidopsis ortholog RAB18 is a useful marker for the loss of ABA sensitivity e.g. in plants that are deficient for the SWI/SNF chromatin-remodeling complex [62]. The gene is upregulated when a relevant repressor is mutated, e.g. SUMO E3 ligase SIZ1 [63]. We hypothesize that the mutant induces a repressor that acts against the ABA pathway or downregulates an inducer of the same pathway. Indeed, two transcription factors were repressed in the mutant under control conditions, the Q-type C2H2 zinc finger transcription factor TaZP23 and the homeobox-leucine zipper protein ATHB-6. *TaZP23* is upregulated in leaves and roots during drought stress and also following ABA treatment, suggesting a role in ABA-dependent gene regulation [64]. ATHB-6 may act as a growth regulator in response to water deficit, and is induced by ABA, salinity and dehydration stress [65]. It also regulates the ABA signaling pathway, acting downstream of ABI1 [66].

The repressed probe sets also included two endonuclease genes related to the barley genes Bnuc and BEN1, which are expressed during apoptosis [67,68]. Bnuc is also induced by salinity stress [69]. The papain-like cysteine proteinase (HvPAP14) and TdS40 were also represented in the repressed probe sets, and these also facilitate senescence and apoptosis [70]. HvPAP14 also generates mature forms of storage proteins in seeds, provides free amino acids during germination [71], and confers stress tolerance and defense against pathogens [72,73]. TdS40 is induced during post-flowering senescence in wheat [74], and also by JA, SA and in chlorotic/necrotic leaf tissue following infection with *Pyrenophora teres*[75].

There were few differences between the mutant and wild-type plants in the absence of water stress at anthesis and no statistically significant differentially-expressed genes could be identified also at the tillering stage (data not shown). Therefore despite a clear difference in chlorophyll content and chloroplast structure, the mutation appears to have a minimal impact on metabolism and gene regulation, a finding that does not provide sufficient explanation for the yield loss detected under field conditions.

### Water stress enhances the differences between mutant and wild-type plants

We investigated the molecular basis of the difference in yield between the two genotypes by exposing them to water stress, which is the most common environmental constraint for durum wheat [4]. Most of the genes modulated by water stress were similarly regulated in both genotypes, suggesting a common response (Additional file 3: Table S2). Genes involved in the synthesis of osmoprotectants were upregulated in both genotypes, whereas genes involved in ABA-dependent signaling, ethylene and JA synthesis were downregulated in both genotypes. Genes related to primary metabolism (amino acid and carbohydrate catabolism) were also similarly modulated, indicating that both genotypes use metabolic strategies to counter the effects of drought. In contrast, genes encoding photosystem components and enzymes representing carbohydrate metabolism and the tricarboxylic acid (TCA) cycle showed significant differences that may explain the difference in performance between wild-type and mutant plants in the field.

#### Photosynthesis and pigment synthesis

Both transcriptomic and proteomic analysis indicated that the components of photosystems I and II (PSI and PSII) and ATPase were differentially expressed in the wild-type and mutant plants under water stress. Several PSI proteins were underrepresented in the mutant, including reaction center (RC) protein subunit IV (PSI-E), which is required for optimal electron transport to ferredoxin and flavodoxin [76], and light harvesting complex LHCA1 (CAB 1B-21) (Table 9). PSI takes longer than PSII to recover from photo-inhibition because RC proteins are degraded instead of being repaired [77]. Therefore, the loss of PSI RC proteins in the mutant is likely to exacerbate the damage caused by stress.

PSBO and PSBP maintain the structure of PSII [78] and were specifically overrepresented in the mutant. CP29 was upregulated in both genotypes, but this was underrepresented in the mutant under stress conditions (Table 9). This protein binds LHCB proteins to the reaction center and confers photoprotection [79]. The LHCB proteins were downregulated in both genotypes and underrepresented in the mutant. In contrast, the genes encoding LHCB proteins were upregulated in the mutant (Table 7). The beta and delta subunits of ATPase synthase were also overrepresented in the mutant, suggesting that the H^+^ flux across the thylakoid membrane is greater in the mutant, with a knock-on effect on ATP production.

Three genes responsible for the synthesis of carotenoid precursors were upregulated by stress specifically in the mutant: 1-deoxy-D-xylulose-5-phosphate synthase (DXS), hydroxymethylglutaryl-CoA synthase (MVA1) and phytoene synthase (PSY). One gene encoding glutamyl-tRNA reductase 1 (HEMA1), which is responsible for the synthesis of chlorophyll precursors, was also upregulated by stress specifically in the mutant. The pigment content of the mutant plants did not increase relative to the control but the activation of these genes could help to prevent a steeper decline in pigment content under water stress conditions (Table 3).

Redox changes in the chloroplast result in the stoichiometric adjustment of photosystem proteins and regulate the expression of both nuclear and plastid photosynthesis-related genes [80-82]. Because the mutant showed evidence of oxidative stress even under control conditions, the re-organization of the photosynthetic apparatus under water stress conditions could reflect the additional oxidative stress that occurs during the early stages of drought. The loss of photosynthetic proteins (especially LHCB) by the mutant in response to drought stress reduces the amount of captured energy and thus avoids the over-excitation of PSII which would generate additional ROS and cause oxidative damage [83].

#### Oxidative stress and ROS signaling

The balance between ROS synthesis and scavenging determines the level of oxidative stress in plants [54,84]. ROS scavenging and detoxifying proteins such as peroxidases, glutathione transferases and glyoxalase were generally modulated in a similar manner in both genotypes, but more probe sets of this group were downregulated specifically in the mutant under stress. In the same direction, a glutaredoxin gene was upregulated in the wild-type plants under stress but was unaffected in the mutant (Table 7). Thylakoid ascorbate peroxidase and catalase were upregulated specifically in the mutant, suggesting a greater need for protection against ROS derived from chloroplast and peroxisome activity (Tables 7 and 9). H_2_O_2_ can spread from the chloroplast to other organelles by diffusion through aquaporin-like PIP2 proteins [85] which were downregulated in the mutant to a lesser extent than in wild-type plants (Additional file 3: Table S2). These results indicate that the mutant experiences greater oxidative stress than wild-type plants under water stress conditions.

Two probe sets related to ROS and stomatal closure were specifically modulated in the mutant under water stress. The specific downregulation of glyoxalase gene suggests that the mutant is less able than wild-type plants to detoxify methylglyoxal using reduced glutathione, thus allowing the production of ROS and stomatal closure [86]. Similarly, the specific upregulation of respiratory burst oxidase homologue D in the mutant (Table 7) suggests that the ABA-induced production of ROS in guard cells is promoted to encourage stomatal closure [87]. However, the differential expression of genes that regulate the stomatal aperture did not result in any differences between the genotypes in terms of stomatal conductance under water stress.

ROS can induce Ca^2+^-dependent signaling that triggers interactions between different calcineurin B-like (CBL) sensors and their CBL-interacting protein kinase (CIPK) targets [88]. Genotype-dependent differences were found in expression of these genes: whereas CIPK3 and CIPK10 were upregulated by stress in both genotypes, CIPK2 and CIPK6 were upregulated specifically in wild-type plants and CBL1 and CIPK23 were downregulated specifically in the mutant. These two proteins cooperate with CBL9 to activate K^+^-channels and regulate stomatal responses [89]. CIPK10 and CIPK6 belong to the SnRK3-type family and mediate nutrient sensing, stress responses and ABA signaling [90,91]. As a result of altered expression of CBLs and CIPKs different Ca^2+^-dependent downstream responses under water stress are expected between mutant and wild-type plants.

MPK3, which was specifically downregulated in the mutant, is also involved in ROS signaling [25]. Once activated, it phosphorylates other stress-related proteins such as the transcription factors ZAT10 and AZF2, and enzymes in the ethylene synthesis pathway such as ACS [92], all of which were downregulated by stress in both genotypes. ZAT10 (STZ) is a transcriptional repressor responsive to chitin, which mediates the response to photo-oxidative stress and ROS [93].

Despite not showing damaged leaves or altered stomatal response, the data suggest that the pale-green mutant differs significantly from wild-type plants in terms of ROS sensing and signaling under water stress. ROS generated in the chloroplast could move more easily inside the cells and elicit a signal triggering specific responses.

#### Carbohydrate metabolism

Transcriptomic profiling revealed differences between the genotypes in the regulation of carbohydrate metabolism under water stress conditions (Table 7). The synthesis of osmoprotectants such as raffinose family oligosaccharides (RFOs) and trehalose was specifically upregulated in the wild-type plants, including the genes encoding galactokinase and raffinose synthases. RFOs protect membranes and scavenge ROS [94]. Trehalose-phosphate synthase (TPS) was also specifically induced in wild-type plants and thus repressed in the mutant under stress. Trehalose and trehalose-6-phosphate act not only as osmolytes to stabilize proteins and membranes, but also as signaling molecules to regulate metabolism.

Two of the probe sets upregulated in the mutant plants encoded sucrose: sucrose 1-fructosyltransferase and sucrose:fructan 6-fructosyltransferase (SST1 and SFT6), which are responsible for synthesizing fructans from sucrose in the vacuole [95,96]. The mutant plants therefore appeared to promote fructan synthesis for remobilization as free carbohydrates, which are directly correlated with the water-soluble carbohydrate content in wheat [96]. Conversely, probe sets encoding cellulose synthase and cellulose synthase-like enzymes were upregulated specifically in the wild-type plants and xylosyltransferases were repressed in the mutant plants under stress (Table 7). These enzymes divert carbon to the synthesis of cell wall polysaccharides (cellulose, hemicellulose and xyloglucans) and their loss reduces growth and also inhibits photosystem activity and carbohydrate metabolism genes [97,98]. This shows the importance of cell wall structure not only for growth and turgor changes under stress but also for carbohydrate metabolism. Furthermore, sucrose, RFOs and fructans can directly or indirectly scavenge ROS, as reviewed by Keunen et al. [99].

The general picture that emerges from the modulation of carbohydrate metabolism suggests that the pale-green mutant is less able to deal with water stress than wild-type plants. Cell wall synthesis genes are activated in the wild-type plants whereas fructans are produced in the mutant. This correlates with the lower biomass of the mutant plants at anthesis and the lower yield under rain-fed conditions (Table 2).

#### TCA cycle and related pathways

The tricarboxylic acid (TCA) cycle generates energy and reducing power (ATP and NADPH) but consumes H_2_O and releases CO_2_. The substrates feeding the cycle are acetyl-CoA and other intermediates from lipid or protein catabolism. Acetyl-CoA is also produced from pyruvate (derived from glycolysis) by the action of the pyruvate dehydrogenase complex, and the subunit E1 gene representing this complex was upregulated in both genotypes under water stress. However, under conditions of carbon starvation or demand, additional strategies are used to supply TCA substrates [100,101]. The wild-type plants appear to be more effective at these strategies given the specific upregulation of the following genes (Table 7):

i) Genes encoding products that convert amino acids into substrates suitable for the TCA cycle [102-104] were upregulated in both genotypes (Additional file 3: Table S2), but three were upregulated specifically in wild-type plants: methylcrotonoyl-CoA carboxylase, α and β subunits (MCCA and MCCB), and 2-oxoisovalerate dehydrogenase E2 (BCE2).

ii) Genes encoding the NADP-malic enzyme (NADP-ME 3 and NADP-ME 4), which converts malate into pyruvate, and phosphoenolpyruvate carboxykinase (PCK1), which converts oxaloacetate into phosphoenolpyruvate, were upregulated specifically in the wild-type plants.

iii) Genes encoding isocitrate lyase (ICL) and malate synthase (MLS) from the glyoxylate cycle were upregulated specifically in wild-type plants and thus, repressed in the mutant. This cycle produces succinate and malate acting as a bypass to avoid the loss of CO_2_ from the normal TCA cycle. It also metabolizes glyoxylate generated by photorespiration and prevents photo-inhibition [105].

iv) Genes encoding glutamate dehydrogenase and glutamate decarboxylase (GDH and GDC) were specifically downregulated and consequently repressed in the mutant under stress. They participate in glutamate metabolism, which balances nitrogen and carbon use in the cell [106] either by storing nitrogen as GABA, glutamate or glutamine, or by supplying carbon back to the TCA cycle. A functional GABA shunt is required for stress tolerance [94]. In the pale-green mutant, carbon stored as glutamate and GABA may not be introduced efficiently into the TCA cycle, disrupting the nitrogen/carbon balance and reducing growth under carbon demand or starvation imposed by stress [107]. GDH can also be inhibited by excess light or sucrose [108].

The feed-in to the TCA cycle and the ability to derive energy from it appear to be more limited in the mutant under stress, potentially explaining the differences between genotypes in terms of biomass and yield in the field experiments. The mutant mobilizes carbon less efficiently, with a significant impact on grain filling and yield as observed in our field experiments.

#### Regulators of carbon metabolism and signaling

Genes that regulate carbohydrate and nitrogen metabolism were also differentially expressed between the wild-type and mutant plants under water stress, helping to explain the basis of the metabolic differences between the genotypes (Table 7).

The *BZIP63* gene was specifically downregulated and repressed in the mutant plants under stress. The transcription factor encoded by this gene is an important node in the glucose-ABA interaction network and is repressed by both ABA and glucose [109]. It may promote starvation tolerance by regulating transcription of genes such as the raffinose synthase (RS6, upregulated in the wild-type plants).

AP2/EREBP proteins mediate hormone, sugar and redox signaling in the context of cold and drought stress [110]. Each member has a unique expression profile and the individual proteins are differentially regulated by glucose [111]. We found that RAP2.12 and RAP2.7 were specifically upregulated in wild-type plants and as a result appeared repressed in the mutant, and that RAP2.8 was specifically downregulated and also appeared repressed in the mutant, suggesting that the mutant and wild-type plants respond differently to the presence of sugars.

PI3P5K was upregulated specifically in the mutant plants. This enzyme increases the levels of phosphatidylinositol-5-phosphate, which functions as a signaling molecule during osmotic stress and can repress the expression of WRKY70 [112]. SAL1, which was downregulated specifically in the mutant, acts as negative regulator of drought tolerance by modulating the levels of different sugars and controlling the corresponding signaling pathways [113].

Since the results highlighted some modifications of signaling molecules, the enzymes of the metabolism could be fine-tuned specifically in each genotype to adjust the concentration of metabolites.

## Conclusions

Under well-watered glasshouse conditions the reduced chlorophyll and carotenoids content of our pale-green mutant resulted on mild oxidative stress symptoms that could be compensated without altering photosynthesis and physiological performance by the induction of stress-response genes. When the availability of water was limited, we observed more extreme modulation of antioxidant enzymes and photosynthetic proteins in the mutant than in wild-type plants, suggesting that the latter were better protected against oxidative stress. Concomitant molecular changes in the mutant revealed that the regulation of carbohydrate metabolism differed from wild-type plants, particularly the energy-producing pathways and pathways feeding the TCA cycle. The modification of carbohydrate metabolism in the mutant resulted in lower biomass accumulation and hence reduced yield under field conditions. This yield reduction was significant (10%) under irrigation, but was exacerbated in rain-fed conditions (14%) in agreement with the gene expression study, thus indicating that the mutant plants were more sensitive to water scarcity. Our results support the conclusion that the pale-green mutant was less able to adapt to terminal water stress, sustaining that reduced pigment content may be disadvantageous in durum wheat under water limited conditions, although a case by case study would be required when working with other pale-green mutants.

## Methods

### Plant material

**Figure 4:**
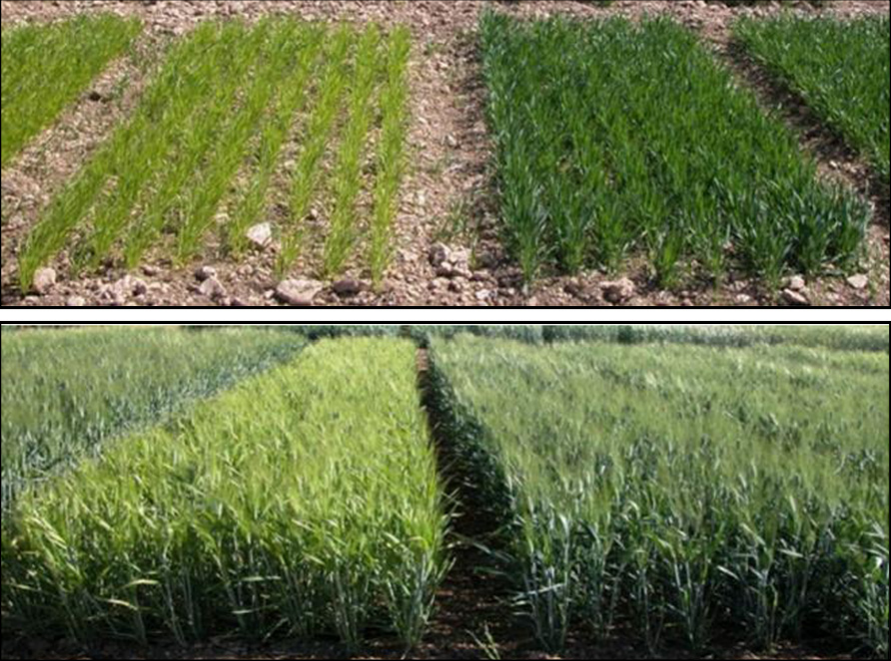
Field plots of the pale-green mutant (MD-597) and the wild-type cv. Borgia.

We compared two durum wheat (*Triticum turgidum* L. var. *durum*) genotypes: the wild-type variety Borgia (from the cross IRTA-1004 x Bidi 17) and a derived pale-green mutant (MD-597). The mutant was selected in 2002 in the M_2_ generation after treating the wild-type variety with the mutagen sodium azide (N_3_Na) and backcrossing twice with the parent to remove other mutations. F_1_ offspring of backcrosses with the wild-type were similar to it, while the ratio of the character segregation was 3:1. Therefore, the mutation is most likely controlled by a recessive nuclear gene.The mutant plants used in our experiments were the stable eighth generation individuals (M_8_) derived from the isolated original mutant plant (Figure 4).

### Experimental treatments

#### Glasshouse experiment

We used a split plot design with three replicates (eight pots per replicate) under controlled conditions in a glasshouse. The main plots were the water treatments (well-watered and water-stressed) and the subplots were the genotypes (wild-type and mutant). Two vernalized seedlings of the same genotype were transplanted into a 4.0-L pots (19 × 15 cm) filled with a blend of white and frozen through black sphagnum peat mixture (Klasmann-Deilmann GmbH), pH 6.5, conductivity 40 ms/m, NPK 14:16:18. Each genotype was planted in 48 pots, giving a total of 96 plants. All plants were grown with the following maximum/minimum temperatures and photoperiods: 13°C/7°C, 12 h daylight from emergence to the first node detectable stage, 18°C/10°C, 14 h daylight from then to heading, 20°C/15°C, 16 h daylight from heading to anthesis, and 25°C/20°C, 18 h daylight after anthesis. The pots were rotated twice a week to avoid temperature and light position effects.

Both genotypes were grown at two levels of soil water content: i) control, in which the soil was maintained at field capacity during the whole experiment, and ii) terminal water stress, in which irrigation was field capacity from sowing to booting (Zadoks stage 45; [114]) and then reduced until maturity by watering the pots every second day with the amount of water necessary to maintain the pots at 1/3 of field capacity. The stress treatment was initiated by withholding water at the anthesis stage. The soil water content was monitored every second day using soil moisture probes (ECH_2_O dielectric aquameter, Decagon Devices, Inc. Pullman, WA, USA) in the center of the pots and was maintained by weighing pots daily and watering them accordingly. Data of the probes indicated that field capacity conditions corresponded to a soil water content around 50 ml water/100 ml of freshweight soil, while the water stress treatment reduced soil water content until ~13 ml water/100 ml soil (Table 1). The flag leaves of four plants per experimental unit were measured between 11 a.m. and 1 p.m. solar time at the booting and anthesis stages to determine (Table 1): i) the midday leaf water potential (Ψ_W_) using a pressure chamber (Model 3005; Soil Moisture Equipment, Santa Barbara, CA); ii) leaf conductance (g_s_) using a steady-state porometer (LI-1600, Li-Cor, Lincoln, NE); and iii) net CO_2_ assimilation rate (An) using a portable infrared gas analyzer (Model LCA-4: Analytical Development, Hoddesdon, UK). For the expression profiling experiments, the flag leaf from five plants was collected for each of three biological replicates and for each water regime, between 11 a.m. and 1 p.m. solar time. The tissues were immediately frozen in liquid nitrogen and stored at -80°C. Each flag leaf was ground in liquid nitrogen and 100-mg and 40-mg aliquots were prepared for transcriptomic and proteomic analysis, respectively. Before RNA and protein extraction, five samples were pooled for each biological replicate.

#### Field experiments

Wild-type and mutant plants were grown in the field for three crop seasons (2010–2012) under irrigated (control) and rain-fed conditions in north-eastern Spain (latitude 41°38’N, longitude 0°23’ E, elevation 250 m a.s.l.) Soil type was Mesic Calcixerolic Xerochrept with a silty-clay texture and pH of 8.1. Plots were sown to a density of 500 seeds/m^2^ in randomized complete block designs with three replicates, each comprising eight rows (12.5 m in length and 15 cm apart). Field conditions are in Table 10. Irrigation was provided four times by flooding the first crop season (on Dec. 8th and at montly intervals from March to May) and by sprinkler irrigations the successive years in which frequencies were determined according to the environmental conditions (Additional file 8: Figure S3). Fertilizer was applied as appropriate, and weeds, diseases and pests were controlled according to standard agricultural practices. Plant biomass was determined at anthesis using a sample of 0.5 m row length and crop dry weight (g/m^2^) following the methodology described by Villegas et al. [115]. Plots were mechanically harvested at ripening and the yield was presented at 12% moisture level.

**Table 10:** Details on field experiments

**Crop season**	**Water regime**	**Sowing date**	**Harvesting date**	**Water input (rainfall** **+** **irrigation, mm)**	**Mean temperature (°C)**	**Mean air relative humidity (%)**
2009-2010	Rainfed	13 Nov. 2009	12 July 2010	287	11.4	70.0
	Irrigated	13 Nov. 2009	13 July 2010	487^a^	11.4	69.9
2010-2011	Rainfed	26 Nov. 2010	5 July 2011	245	11.5	70.3
	Irrigated	27 Nov. 2010	7 July 2011	395^b^	11.7	70.2
2011-2012	Rainfed	9 Dec. 2011	2 July 2012	160	11.5	62.8
	Irrigated	8 Dec. 2011	12 July 2012	335^b^	12.0	62.9

^a^Flooding irrigation.

^b^Sprinkler irrigation.

### Microscopy

Three plantlets at the three fully-developed leaves stage (Zadoks stage 13; [114]) from the glasshouse experiment, irrigated at field capacity, were collected from each genotype. Small segments (1 mm) from the central part of the third-leaf blades were fixed in 2.5% glutaraldehyde in 0.1 M phosphate buffer (pH 7.2) at 4°C. After three washes in 0.1 M phosphate buffer (pH 7.4) the samples were incubated in 1% osmium tetroxide and 2.5% potassium ferrocyanide for 2 h at 4°C. After ten washes for 10 min each with 0.1 M sodium acetate, and incubation for 30 min in 0.5% uranyl acetate followed by two washes for 10 min in 0.1 M sodium acetate at 4°C, the samples were dehydrated in increasing concentrations of ethanol followed by two washes with sodium acetate and finally embedded in epoxy EMBED-812 resin (Electron Microscopy Sciences) for three days at 60°C. Ultrathin sections (70 nm) contrasted with Reynolds’ lead citrate stain were examined using a Zeiss TEM 910 transmission electron microscope.

### Pigment measurements

Pigments were quantified in four wild-type and mutant plants from each treatment regime harvested at anthesis. The blade of the flag leaf was dissected, weighed and immediately frozen in liquid nitrogen. The pigments from 100 mg lyophilized tissue were extracted with acetone for 20 min at 40°C, filtered and mixed with 9:1 hexane:ethyl ether and 2% NaCl. The organic fraction was evaporated in a nitrogen stream and argon was used for storage at -80°C. The pigments were separated on an ACQUITY Ultra Performance LC™ system (Waters, Milford, MA, USA) linked to a PDA 2996 detector (Waters, Milford, MA, USA) following the protocol of Taylor et al. [116] but using a modified gradient.

Carotenoids were isolated using a YMC C_30_ carotenoid 3-μm, 2.0 × 100 mm HPLC column (Waters, Milford, MA) with a mobile phase comprising solvent A (methanol:water, 97:3, v/v) and solvent B (100% methyl *tert*-butylether), both solvents containing 0.05% triethylamine. The sample was warmed to 25°C and a volume of 10 μL was injected into the column, which also was thermostatically maintained at 25°C. The flow rate was set to 0.5 mL/min. The gradient program was set as follows: initial conditions 98% solvent A and 2% solvent B for 13 min; changed with a linear gradient to 62% solvent A and 38% solvent B in 1 min; hold for 2 min; changed with a linear gradient to 32% solvent A and 68% solvent B in 1 min; hold for 5 min; returned to initial conditions in 4 min, followed by equilibration for 5 min. The data analysis was done with MassLynx™ software version 4.1 (Waters, Milford, MA, USA). Carotenoids were identified by monitoring the order of elution from the column, the ultraviolet and visible spectra and the spectral fine structure and comparing them with standards and previous references [117]. Standards trans-β-apo-8’carotenal, chlorophyll *a* and chlorophyll *b* were purchased from Sigma-Aldrich (USA), whereas lutein (L), zeaxanthin (Z), β-carotene (β-car), neoxanthin (N), violaxanthin (V) and antheraxanthin (A) were acquired from CaroteNature (Switzerland). The data were analyzed by ANOVA with the SAS-STAT package and the means were compared with a Tukey test at 5% probability level.

### RNA isolation and array hybridization

Total RNA was extracted using Trizol reagent (Invitrogen, Carlsbad, CA, USA) and cleaned with RNeasy MinElute (QIAGEN) columns following the manufacturer’s instructions. Quality was determined using an Agilent Bioanalyzer 2100. The RNA samples were processed following the Affymetrix Genechip Expression Analysis Technical Manual. Single-stranded and double-stranded cDNAs were synthesized using the Affymetrix GeneChip® 3’ IVT Express Kit. Biotin-conjugated nucleotides were incorporated into aRNA by *in vitro* transcription to generate cRNA which was purified to remove unincorporated NTPs, salts, enzymes and inorganic phosphate before fragmentation and hybridization onto 3’ expression arrays. Specifically, we used the Affymetrix GeneChip® Wheat Genome Array. The arrays were washed and stained on an Affymetrix Fluidics Station followed by scanning with a GeneChip Scanner 3000.

### GeneChip® quality analysis

The quality of microarray hybridizations was determined by checking the following parameters: i) the standard Affymetrix controls to evaluate labeling and hybridization (B2 oligonucleotides, PolyA controls such as *lys*, *phe*, *thr* and *dap*, and hybridization controls such as *BioB*, *BioC*, *BioD* and *Cre*); ii) RNA degradation plot relative to probe signal intensities of the *Actin* and *GAPDH* control genes, using the R library "simpleaffy"; iii) percentage of "Present calls" on the basis of the MAS 5.0 algorithm; and iv) R^2^ linear correlation coefficients among biological.

### Data processing and analysis

Hybridization data were processed as recommended by Aprile et al. [118]: i) normalization of the raw data by Robust Multi-Array Average (RMA) [119] using the R package Affymetrix library [120]; ii) MAS 5.0 algorithm on raw data in order to produce a detection call for each probe set "Present", "Marginal" or "Absent" (all probe sets that did not show a "Present" call in all repetitions of at least one sample were removed); iii) the filtered data were imported into the software Genespring GX7.3 (Agilent) for the analysis of differentially-expressed genes using a Welch *t*-test with Benjamini and Hochberg false discovery rate correction for multiple tests [121].

The differences in gene expression were considered to be significant at *P* < 0.05 and the induction or repression ratio was ≥2-fold. Principal component analysis (PCA) was then used to validate the role of the genotype and to verify the effect of water stress between the wild-type and mutant plants (Figure 2).

The terms induced or repressed were used when describing the comparison on gene expression between both genotypes independently of the water treatment (mutation effect). And the up/downregulated terms were used when comparing the two water treatments in the same genotype (stress effect).

In order to identify overrepresented gene classes within selected groups of genes compared to the entire array, the *Arabidopsis thaliana* best BLASTX annotations of the wheat probe sets were used as input for the MIPS Functional Catalogue Database (FunCatDB) [122], which provides a statistical survey of the functional distribution of a given set of genes. Only the functional categories with a cut-off of *P* < 0.005 were considered. BLAST searches were carried out using Plant Expression Database (PLEXdb) [123] where a complete list of additional annotation for all probe sets on the wheat genome array is available. The original microarray data have been deposited and are accessible in PLEXdb ( http://www.plexdb.org) as experiment TA48: "Pale-green durum wheat mutant under terminal drought stress condition" and in NCBI's Gene Expression Omnibus (NCBI GEO; http://www.ncbi.nlm.nih.gov/geo) as experiment GSE45563.

### Validation of array data with qRT-PCR

Quantitative real-time RT-PCR (qRT-PCR) was used to confirm the differential expression of the probe sets identified by microarray analysis. The total RNA isolated for the array experiment was treated with DNA-free™ DNase Treatment & Removal Reagents (Ambion, Life technologies) and cDNA was synthesized using the iScript cDNA synthesis kit (Bio-Rad, Hercules, CA, USA). PCR amplification was carried out in a 25 μL final volume containing 2× IQ SYBR Green Supermix (BioRad, Hercules, CA, USA), 500 nM of each specific forward and reverse primers and 10 ng of cDNA as the template, using the Bio-Rad CFX96 sequence detector system. The amplification program comprised a denaturation step at 95˚C for 3 min followed by 40 cycles of 95˚C for 10 s, 60˚C for 30 s and 72˚C for 20 s. The amplification products were validated by melt curve analysis using standard curves with an efficiency of 90–100%. Several repetitions were used to establish the efficiency of the standard curve for the internal reference gene. Efficiency was auto-calculated using the Bio-Rad CFX Manager software [124]. Ct value and normalized expression were calculated using the ΔΔC(t) method [125]. The no reverse transcriptase and no template negative controls were used to ensure absence of contamination and of nonspecific amplifications. Primer specificity was further confirmed by gel electrophoresis.

Specific qRT-PCR were used to verify nine probe sets corresponding to the following genes: *Chitinase 3* (TaAffx.128418.43.S1_at), *Blufensin 1* (TaAffx.26815.1.S1_at), *Germin*-*like protein* (Ta.169.1.S1_x_at), *Naringenin*,*2*-*oxoglutarate 3*-*dioxygenase* (TaAffx.6092.1.S1_at), *WRKY45*-*like transcription factor* (Ta.8614.2.S1_x_at), *Agmatine coumaroyltransferase* (Ta.8228.1.S1_at), RAB protein (Ta.2638.1.S1_at), *Q*-*type C2H2 zinc finger protein* (*ZFP23*) (TaAffx.98394.1.S1_at) and *Dehydrin* (*WZY1*-*1*; Ta.13255.1.S1_at). Primers were designed near the 3’, according to GenBank (NCBI) database sequences (Additional file 9: Table S6). Data were calculated from the calibration curve and normalized using the expression curve of the *Actin* transcript (GQ339780) corresponding to probe set Ta.28253.1.S1_at, selected among several probe sets with low variation between samples as the internal reference gene (CV = 0.036). The qRT-PCR data were compared to the corresponding microarray expression values by mean of Pearson product–moment correlation coefficients (Additional file 1: Figure S1).

### Protein extraction

Protein was extracted as described by Jiang et al. [126] with modifications. The powder was resuspended in 2 mL ice-cold 10% trichloroacetic acid in acetone containing 0.07% dithiothreitol (DTT) and protease inhibitor cocktail (P9599 Sigma). After overnight incubation at -20°C, the mixture was centrifuged at 4 °C for 30 min at 14000 rpm. The pellet was washed four times with acetone containing 0.07% DTT and centrifuged for 5 min at 14000 rpm. The pellet was left to stand on ice until the acetone evaporated completely. Protein Extraction Reagent type 4 buffer (C0356 Sigma) was used to resuspend the pellet overnight at room temperature while mixing. Proteins were separated from insoluble components by centrifugation for 20 min at 14000 rpm and were stored at -80°C. The Bradford protein quantification method [127] was used to quantify the proteins using bovine serum albumin as a standard.

### 2-D Electrophoresis and detection

For the first dimension separation, samples were mixed with rehydration buffer (7 M urea, 2 M thiourea, 1% C7BzO detergent, 40 mM Trizmabase, 50 mM DTT, 1% IPG buffer pH 3–10, and 0.002% bromophenol blue) to a total volume of 315 μL. Actively rehydrated (50 V/gel, 20˚C) 18-cm Bio-Rad IPG strips (pH 5–8) were loaded with protein and separated by isoelectric focusing using a Protean IEF Cell system (Bio-Rad) following the manufacturer’s instructions. The sequential gradient procedure was 500 V/linear for 30 min, 1000 V/linear for 1 h, 10000 V/linear for 1 h, and 10000 V/rapid until a total of 55,000 Vh. The current limit was 50 μA per IPG strip. After IEF separation, the gel strips were incubated for 15 min in the equilibration buffer (375 mM Tris base, 6 M urea, 20% glycerol, 2% SDS) containing 2% DTT, followed by 15 min in the same buffer containing 2.5% iodoacetamide instead of DTT. For the second-dimension separation, SDS-PAGE with 11% 22 × 20 cm polyacrylamide gels were loaded and separated in an Ettan DALTsix Electrophoresis Unit (GE Healthcare) with 0.25 M Tris–HCl (pH 8.8), 1.92 M glycine, 1% w/v SDS electrophoresis buffer, at 8 mA/gel overnight. Three replicate analytical gels, with 50 μg of proteins each, were run for each protein extract. The preparative gels were loaded with 350 μg of protein. After electrophoresis, the analytical and preparative SDS-PAGE gels were fluorescently stained with Flamingo or Oriole (Bio-Rad) respectively, and scanned with a Versadoc MP 4000 system (Bio-Rad).

### Image analysis

Gel imaging and spot quantification were carried out using the PD-Quest Advanced 2D Gel Analysis software v8.0.1 (Bio-Rad) applying automatic spot detection and matching followed by manual/visual validation. The image was normalized by local regression and a scaling factor of 10^6^ (ppm) was applied to avoid non-expression related variations in spot intensity. Spots showing more than 2-fold changes in intensity with a coefficient of variation <50% and with statistically significant differences by Student’s t-test (*P* < 0.05) were defined as differentially expressed.

### Protein digestion and identification by mass spectrometry (MS)

The spots of interest were excised manually from preparative gels and digested with trypsin according to Perez-Hedó et al. [128]. We then applied 1 μL of tryptic peptide solution to a MALDI plate, dried it at room temperature and covered it with 1 μL of saturated α-cyano-4-hydroxycinnamic acid prepared in 50% v/v ACN containing 0.1% TFA.

Proteins were identified using an AutoflexSpeed MALDI-TOF/TOF mass spectrometer (Bruker Daltonics) by peptide mass fingerprinting (PMF). For those spots lacking identification, 3–4 peptides were analyzed by MS/MS mode. Mass spectra (mode reflectron, MH^+^) were acquired by FlexControl v3.0 software (Bruker Daltonics), recording in the range 800–4500 Da, and the MS/MS information was obtained in LIFT (Laser-Induced Forward Transfer) mode. MS spectra were externally calibrated using Peptide Calibration Standard II (Bruker Daltonics). The peak lists obtained were compared against the Swiss-Prot, Trembl and non-redundant NCBI protein databases, using the MASCOT software package v2.3 (Matrix Sciences, UK). The search parameters were set as monoisotopic peptide masses, carbamidomethylation of cysteine and oxidation of methionine as fixed and variable modifications, respectively, one trypsin missed cleavage and a maximum of ±100 ppm for PMF peptide tolerance and ±0.4 Da for MS/MS tolerance. Protein identification was accepted for those results showing a probability-based MOWSE score with *P* < 0.05. We also analyzed MS and MS/MS combined spectra prepared using BioToolsv3.1 (Bruker Daltonics).

### Availability of supporting data

The data set supporting the results of this article is available in the PLEXdb repository as experiment TA48.

## Abbreviations

ABA: Abscisic acid; ACS: 1-aminocyclopropane-1-carboxylate synthase; CAB: Chlorophyll binding protein; CBL: Calcineurin B-like; CIPK: CBL-interacting protein kinase; GABA: γ-aminobutyric acid; GDC: Glutamate decarboxylase; GDH: Glutamate dehydrogenase; JA: Jasmonate; LEA: Late embryogenesis abundant protein; LHC: Light harvesting complex; PI3P5K: phosphatidylinositol-3-phosphate 5-kinase; PR: Pathogenesis-related; PS: Photosystem; PSBO: Photosystem subunit O; PSBP: Photosystem subunit P; RAB: Responsive to ABA; RAP: Related to AP2; RC: Reaction center; RFO: Raffinose family oligosaccharides; ROS: Reactive oxygen species; SA: Salicylic acid; SWC: Soil water content; TCA: Tricarboxylic acid.

## Competing interests

The authors declare that they have no competing interests.

## Authors’ contributions

AP carried out the molecular analysis and drafted the manuscript. CM participated in the transcriptomic analysis and interpretation of data. AA carried out the statistical analysis of transcriptomic data. ER carried out the microarray hybridization. CR, LC and DV helped to draft the manuscript and interpretation of data. CR released the mother variety and conceived the study. CR and DV obtained the mutant. All authors read and approved the final manuscript.

## Supplementary Material

Additional file 1: Figure S1 Relationship between qRT-PCR and microarray expression data. Click here for file

Additional file 2: Table S1 Significant functional categories represented in the probe sets modulated similarly in the wild-type and mutant plants at anthesis under water stress conditions. Click here for file

Additional file 3: Table S2 Selected probe sets modulated similarly in the wild-type and mutant plants representing important processes occurring under water stress conditions. Click here for file

Additional file 4: Table S3 Significant functional categories represented in the induced and repressed probe sets in the mutant plants under water stress conditions at anthesis. Click here for file

Additional file 5: Figure S2 2D-gel electrophoresis of leaf proteins from wild-type and mutant plants under water stress. Click here for file

Additional file 6: Table S4 Protein identification in differently expressed spots by peptide mass fingerprinting (PMF). Click here for file

Additional file 7: Table S5 Protein identification in differently expressed spots by MS/MS analysis. Click here for file

Additional file 8: Figure S3 Rain and irrigation pattern in the field experiments. Click here for file

Additional file 9: Table S6 Sequence of primers used for qRT-PCR. Click here for file
