# Supplementary material for: Transcriptomic and proteomic analyses of a pale-green durum wheat mutant shows variations in photosystem components and metabolic deficiencies under drought stress
Source: BMC Genomics. 2014 Feb 12;15:125. doi: 10.1186/1471-2164-15-125 (PMC3937041; doi:10.1186/1471-2164-15-125)

**Additional file figure 1.** Relationship between qRT-PCR and microarray expression data in the mutant (M) *versus* the wild-type (WT) under control conditions. The Pearson product-moment correlation coefficient is 0.96.


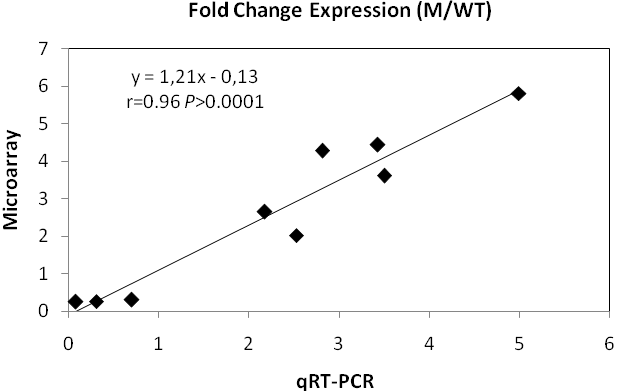

Supplement: Additional file 1: Figure S1 — Relationship between qRT-PCR and microarray expression data. [file 1471-2164-15-125-S1.doc]
