# Supplementary material for: Transcriptomic and proteomic analyses of a pale-green durum wheat mutant shows variations in photosystem components and metabolic deficiencies under drought stress
Source: BMC Genomics. 2014 Feb 12;15:125. doi: 10.1186/1471-2164-15-125 (PMC3937041; doi:10.1186/1471-2164-15-125)
Supplement: Additional file 2: Table S1 — Significant functional categories represented in the probe sets modulated similarly in the wild-type and mutant plants at anthesis under water stress conditions. [file 1471-2164-15-125-S2.doc]

**Additional file table 1.** Significant functional categories represented in the probe sets modulated similarly in the wild-type and mutant plants at anthesis under water stress conditions according to the MIPS Functional Catalogue Database (Ruepp et al., 2004). Only functional categories with a cut-off of *P* ≤ 0.005 were considered.

| **Upregulated** | Number of genes | *P*-value |
| --- | --- | --- |
| 01 METABOLISM | 61 | 5.30E-09 |
| 01.01 amino acid metabolism | 10 | 4.34E-05 |
| 01.01.03 assimilation of ammonia, metabolism of the glutamate group | 3 | 4.86E-03 |
| 01.01.03.03 metabolism of proline | 2 | 1.55E-03 |
| 01.01.03.03.01 biosynthesis of proline | 2 | 3.52E-04 |
| 01.01.06 metabolism of the aspartate family | 4 | 1.79E-03 |
| 01.01.06.06.01 biosynthesis of lysine | 2 | 4.56E-03 |
| 01.01.11 metabolism of the pyruvate family (alanine, isoleucine, leucine, valine) and D-alanine | 3 | 2.18E-03 |
| 01.01.11.02 metabolism of isoleucine | 2 | 3.55E-03 |
| 01.01.11.03 metabolism of valine | 2 | 4.56E-03 |
| 01.05 C-compound and carbohydrate metabolism | 26 | 5.59E-06 |
| 01.05.02 sugar, glucoside, polyol and carboxylate metabolism | 16 | 5.76E-05 |
| 16.09 lipid binding | 8 | 2.24E-06 |
| 20 CELLULAR TRANSPORT, TRANSPORT FACILITIES AND TRANSPORT ROUTES | 31 | 4.14E-05 |
| 20.01 transported compounds (substrates) | 29 | 2.70E-06 |
| 20.01.01.07.07 phosphate transport | 2 | 4.56E-03 |
| 20.01.13 lipid/fatty acid transport | 8 | 7.99E-07 |
| 32 CELL RESCUE, DEFENSE AND VIRULENCE | 23 | 1.46E-05 |
| 32.01 stress response | 19 | 6.55E-07 |
| 32.01.01 oxidative stress response | 8 | 2.90E-05 |
| 32.01.05 heat shock response | 5 | 1.61E-04 |
| 32.07.07.05 peroxidase reaction | 4 | 2.48E-03 |
| 34 INTERACTION WITH THE ENVIRONMENT | 21 | 9.02E-04 |
| 34.11 cellular sensing and response to external stimulus | 21 | 2.35E-04 |
| 34.11.03 chemoperception and response | 14 | 5.61E-04 |
| 34.11.09 temperature perception and response | 8 | 1.95E-04 |
| 36 SYSTEMIC INTERACTION WITH THE ENVIRONMENT | 14 | 1.96E-04 |
| 36.20 plant / fungal specific systemic sensing and response | 12 | 9.83E-04 |
| 36.20.18 plant hormonal regulation | 12 | 2.37E-04 |
| 36.20.18.05 abscisic acid response | 7 | 5.46E-05 |
| 40 CELL FATE | 10 | 4.02E-04 |
| 40.20 cell aging | 5 | 1.11E-05 |
| 43 CELL TYPE DIFFERENTIATION | 6 | 2.06E-03 |
| 43.02 plant cell type differentiation | 5 | 4.54E-03 |
| 70.01 cell wall | 8 | 4.57E-03 |
| 77.02.01.02 leaf | 2 | 3.09E-03 |

| **Downregulated** | Number of genes | *P*-value |
| --- | --- | --- |
| 01 METABOLISM | 106 | 1.22E-16 |
| 01.01 amino acid metabolism | 11 | 5.97E-04 |
| 01.01.09 metabolism of the cysteine - aromatic group | 7 | 3.97E-04 |
| 01.01.09.04 metabolism of phenylalanine | 5 | 1.64E-05 |
| 01.04 phosphate metabolism | 52 | 6.21E-12 |
| 01.20 secondary metabolism | 24 | 7.04E-12 |
| 01.20.05 metabolism of acetic acid derivatives | 4 | 6.86E-05 |
| 01.20.35 metabolism of secondary products derived from L-phenylalanine and L-tyrosine | 8 | 4.17E-05 |
| 01.20.35.01 metabolism of phenylpropanoids | 7 | 1.58E-04 |
| 11.02.03.04.03 transcription repression | 4 | 2.36E-03 |
| 14 PROTEIN FATE (folding, modification, destination) | 46 | 2.79E-03 |
| 14.07 protein modification | 36 | 1.27E-03 |
| 14.07.03 modification by phosphorylation, dephosphorylation, autophosphorylation | 30 | 5.48E-06 |
| 16 PROTEIN WITH BINDING FUNCTION OR COFACTOR REQUIREMENT (structural or catalytic) | 93 | 6.41E-04 |
| 16.17 metal binding | 27 | 1.95E-04 |
| 16.17.01 calcium binding | 7 | 1.63E-03 |
| 16.25 oxygen binding | 13 | 6.30E-07 |
| 20.01 transported compounds (substrates) | 31 | 3.46E-03 |
| 20.01.15 electron transport | 15 | 4.98E-03 |
| 30.01.05.01.06 serine/threonine kinase | 7 | 2.31E-03 |
| 32 CELL RESCUE, DEFENSE AND VIRULENCE | 39 | 6.70E-09 |
| 32.01 stress response | 26 | 2.25E-07 |
| 32.01.01 oxidative stress response | 11 | 4.90E-06 |
| 32.01.06 cold shock response | 9 | 3.82E-05 |
| 32.05 disease, virulence and defense | 13 | 6.61E-04 |
| 32.07 detoxification | 10 | 3.15E-04 |
| 32.07.07 oxygen and radical detoxification | 10 | 2.71E-04 |
| 32.07.07.03 glutathione conjugation reaction | 4 | 2.36E-03 |
| 32.07.07.05 peroxidase reaction | 5 | 2.34E-03 |
| 34 INTERACTION WITH THE ENVIRONMENT | 43 | 4.44E-09 |
| 34.11 cellular sensing and response to external stimulus | 43 | 1.95E-10 |
| 34.11.03 chemoperception and response | 26 | 2.76E-07 |
| 34.11.09 temperature perception and response | 11 | 6.21E-05 |
| 34.11.10 response to biotic stimulus | 11 | 1.76E-04 |
| 36 SYSTEMIC INTERACTION WITH THE ENVIRONMENT | 31 | 2.15E-11 |
| 36.20 plant / fungal specific systemic sensing and response | 26 | 5.83E-09 |
| 36.20.16 plant defense response | 7 | 2.26E-04 |
| 36.20.16.03 jasmonic acid/ethylene dependent systemic resistance | 3 | 1.82E-03 |
| 36.20.18 plant hormonal regulation | 22 | 1.00E-07 |
| 36.20.18.05 abscisic acid response | 8 | 1.96E-04 |
| 36.20.18.99 other plant signalling molecules response (jasmonic acid, salicylic acid etc.) | 10 | 2.64E-05 |
| 36.25 animal specific systemic sensing and response | 8 | 1.07E-05 |
| 36.25.16 immune response | 8 | 6.49E-06 |
| 36.25.16.08 response to wounding | 7 | 1.37E-05 |
| 70.02 eukaryotic plasma membrane / membrane attached | 16 | 5.71E-05 |
