# Supplementary material for: Transcriptomic and proteomic analyses of a pale-green durum wheat mutant shows variations in photosystem components and metabolic deficiencies under drought stress
Source: BMC Genomics. 2014 Feb 12;15:125. doi: 10.1186/1471-2164-15-125 (PMC3937041; doi:10.1186/1471-2164-15-125)
Supplement: Additional file 3: Table S2 — Selected probe sets modulated similarly in the wild-type and mutant plants representing important processes occurring under water stress conditions. [file 1471-2164-15-125-S3.doc]

**Additional file table 2.** Selected probe sets modulated similarly in the wild-type and mutant plants representing important processes occurring under water stress conditions. Last column (M *vs* WT) compares mutant against wild-type expression under water stress. Positive and negative values indicate upregulation and downregulation, respectively, whereas no statistically significant differences are indicated with a dash (-).

| Probe set ID | Gene Name | Arabidopsis ID | Log_2_ ratio WT stress/WT control | Log_2_ ratio M stress/M control | Log_2_ ratio M/WT under water stres |
| --- | --- | --- | --- | --- | --- |
| **Carbohydrate metabolism** | | | | | |
| Ta.11152.3.S1_at | SUS3; Sucrose synthase 3 | AT4G02280 | 2.02 | 1.92 | - |
| Ta.11152.2.A1_a_at |  |  | 1.92 | 1.95 | - |
| Ta.93.1.S1_at | SUS4; Sucrose synthase 4 | AT3G43190 | 1.33 | 1.41 | - |
| TaAffx.38560.1.A1_at | G6PD4; Glucose-6-phosphate dehydrogenase 4 | AT1G09420 | 1.58 | 1.46 | - |
| Ta.277.1.S1_at | ATLDA (LIMIT DEXTRINASE); Alpha-amylase/ limitdextrinase/ pullulanase | AT5G04360 | 1.83 | 1.83 | - |
| Ta.9641.1.A1_a_at | Fructan 6-exohydrolase | Q2UXF7* | 3.03 | 4.79 | - |
| Ta.30950.1.A1_x_at |  |  | 2.83 | 4.10 | - |
| Ta.26928.1.S1_a_at | Fructan exohydrolase | Q3MV21* | 1.35 | 1.54 | - |
| Ta.26928.1.S1_x_at |  |  | 1.09 | 1.24 | - |
| Ta.26928.2.S1_x_at |  |  | 1.25 | 1.28 | - |
| TaAffx.16032.1.A1_at | CWINV2; Cell wall invertase 2 | AT3G52600 | -1.18 | -1.43 | - |
| Ta.1042.1.S1_x_at | AGAL2; Alpha-galactosidase 2 | AT5G08370 | 2.24 | 1.74 | - |
| Ta.21792.1.S1_s_at | AGLU1; Alpha-glucosidase1 | AT5G11720 | 1.08 | 1.18 | - |
| TaAffx.79139.1.S1_at | ICL; Isocitrate lyase | AT3G21720 | 3.08 | 2.24 | -1.90 |
| Ta.6144.1.S1_a_at | TPPD; Trehalose-6-phosphate phosphatase D | AT1G35910 | -1.31 | -1.25 | - |
| Ta.6144.2.S1_a_at |  |  | -1.29 | -1.37 | - |
| Ta.10237.1.S1_at | TPPF; Trehalose-6-phosphate phosphatase F | AT4G12430 | -1.01 | -1.04 | - |
| **Amino acid metabolism** | | | | | |
| TaAffx.95414.1.S1_at | BCAT3; Branched-chain-amino-acid aminotransferase 3 | AT3G49680 | 1.51 | 1.77 | - |
| Ta.29508.3.S1_a_at | BCKDHE1α; Branched-chain alpha-keto acid dehydrogenase E1 alpha subunit | AT5G09300 | 1.38 | 1.05 | - |
| Ta.28675.1.A1_at | AK3; Aspartokinase 3 | AT3G02020 | 1.27 | 1.21 | - |
| Ta.4834.1.S1_at | PYD4; Pyrimidine 4; alanine-glyoxylate transaminase | AT3G08860 | 1.79 | 1.52 | - |
| TaAffx.12940.1.A1_at | crr1; Putative dihydrodipicolinate reductase 3 | AT5G52100 | 1.16 | 1.51 | 1.03 |
| Ta.4800.1.S1_at | LKR/SDH, Lysine-ketoglutarate reductase/Saccharopine dehydrogenase | AT4G33150 | 1.44 | 1.10 | - |
| Ta.8615.2.S1_at | THA2; Threonine aldolase 2 | AT3G04520 | -0.97 | -2.24 | - |
| **Proline metabolism** | | | | | |
| Ta.7091.1.S1_at | P5CS2; Gamma-glutamyl phosphate reductase | AT3G55610 | 3.11 | 2.79 | - |
| Ta.591.1.S1_at | P5CR; Pyrroline-5-carboxylate reductase | AT5G14800 | 1.21 | 1.06 | - |
| **ABA response** | | | | | |
| Ta.21082.2.S1_at | RCAR8; Regulatory component of ABA receptor 8;PYR1-LIKE 5 (PYL5) | AT5G05440 | -1.40 | -2.43 | -1.17 |
| Ta.21082.2.S1_s_at | RCAR10; Regulatory component of ABA receptor 10;PYR1-LIKE 4 (PYL4) | AT2G38310 | -1.41 | -2.19 | - |
| Ta.10098.1.S1_at | PP2C; Protein phosphatase 2C, putative | AT2G25620 | -1.05 | -1.56 | - |
| Ta.10098.1.S1_x_at |  |  | -1.10 | -1.24 | - |
| TaAffx.16090.1.S1_at | PP2C; Protein phosphatase 2C family protein | AT4G33920 | -1.42 | -1.47 | - |
| Ta.13255.1.S1_at | RAB18; Responsive to ABA 18 | AT5G66400 | 1.27 | 2.79 | - |
| Ta.2638.1.S1_at |  |  | 1.36 | 2.60 | - |
| Ta.29352.1.S1_at |  |  | 1.94 | 1.65 | - |
| Ta.29352.1.S1_x_at |  |  | 2.49 | 2.79 | - |
| TaAffx.46097.1.S1_at |  |  | 1.86 | 1.32 | - |
| Ta.28209.2.S1_x_at | RD22; Responsive to dessication 22 | AT5G25610 | 1.05 | 1.72 | - |
| **Jasmonic Acid** | | | | | |
| Ta.1207.1.S1_at | OPR1; 12-oxophytodienoate reductase | AT1G76680 | -1.32 | -1.60 | - |
| Ta.1207.1.S1_s_at |  |  | -1.27 | -1.30 | - |
| Ta.1207.1.S1_x_at |  |  | -1.30 | -1.33 | - |
| TaAffx.128684.1.S1_at |  |  | -1.59 | -1.62 | - |
| TaAffx.128684.1.S1_x_at |  |  | -1.60 | -2.20 | - |
| Ta.5509.1.S1_at | OPR2; 12-oxophytodienoate reductase | AT1G76690 | -1.25 | -1.77 | - |
| Ta.5149.1.A1_at | JAZ11; Jasmonate-ZIM-domain protein 11 | AT3G43440 | -2.11 | -1.41 | - |
| Ta.13362.1.S1_at | AOS; Allene oxide synthase | AT5G42650 | -1.56 | -1.42 | - |
| Ta.23763.1.S1_at | LOX1; Lipoxygenase | AT1G55020 | -2.35 | -1.64 | - |
| **Ethylene** | | | | | |
| Ta.9107.1.S1_x_at | EFE (Ethylene-Forming Enzyme); 1-aminocyclopropane-1-carboxylate oxidase | AT1G05010 | 1.99 | 1.82 | - |
| Ta.9107.2.S1_a_at |  |  | 2.49 | 2.19 | - |
| Ta.9107.2.S1_at |  |  | 2.19 | 2.01 | - |
| TaAffx.100446.1.S1_at |  |  | 3.33 | 3.22 | - |
| Ta.25755.1.A1_at | ACS2; 1-aminocyclopropane-1-carboxylate synthase | AT1G01480 | -1.81 | -2.22 | - |
| Ta.14000.1.S1_at | ERF4; Ethylene Responsive Element Binding Factor 4 | AT3G15210 | -1.09 | -1.24 | - |
| **Ca^2+^Signaling** | | | | | |
| Ta.28002.1.A1_at | CIPK3; CBL-Interacting protein kinase 3 | AT2G26980 | 1.98 | 2.26 | - |
| Ta.28002.2.A1_a_at |  |  | 2.31 | 2.53 | - |
| Ta.25609.1.S1_at | CIPK10; CBL-Interacting protein kinase 10; SIP1 (SOS3-Interacting protein 1) | AT5G58380 | 1.83 | 1.47 | - |
| Ta.5272.2.S1_a_at | CBL1; Calcineurin B-like protein 1 | AT4G17615 | -1.06 | -1.33 | - |
| **Transcription** | | | | | |
| Ta.5433.1.S1_a_at | WRKY31 | AT4G22070 | -1.00 | -1.35 | - |
| Ta.4725.1.S1_at | WRKY33 | AT2G38470 | -2.01 | -1.95 | - |
| Ta.4678.1.S1_at | WRKY40 | AT1G80840 | -1.20 | -1.98 | - |
| Ta.4678.1.S1_x_a |  |  | -1.19 | -2.02 | - |
| Ta.4678.2.S1_at |  |  | -1.30 | -1.97 | - |
| Ta.16082.1.A1_a_at | WRKY41 | AT4G11070 | -1.39 | -1.15 | - |
| Ta.8614.1.S1_at | WRKY70 | AT3G56400 | -1.03 | -2.02 | - |
| Ta.29449.1.S1_s_at | STZ; Salt tolerance zinc finger | AT1G27730 | -1.16 | -1.44 | - |
| **Water channels** | | | | | |
| TaAffx.8804.1.S1_s_at | PIP2;2; Plasma Membrane Intrinsic Protein 2;2; water channel | AT2G37170 | -2.65 | -1.69 | 1.07 |
| TaAffx.8804.3.S1_s_at |  |  | -3.17 | -1.95 | 1.36 |
| TaAffx.8804.2.S1_at | PIP2;5; Plasma Membrane Intrinsic Protein 2;5; water channel | AT3G54820 | -2.09 | -1.17 | 1.04 |
| **Others** | | | | | |
| Ta.2631.1.S1_at | MIOX1; *myo*-Inositol oxygenase | AT1G14520 | 1.21 | 2.22 | 1.17 |
| Ta.13232.1.S1_at | SMO1-2; Sterol C4-methyl oxidase 1-2 | AT4G22756 | 2.30 | 1.57 | 0.83 |
| Ta.2107.1.S1_s_at | ALDH7B4; Aldehyde dehydrogenase 7B4 | AT1G54100 | 2.17 | 1.11 | - |
| Ta.2107.3.S1_at |  |  | 2.16 | 1.06 | -1.18 |
| *UniProt ID from *Triticum aestivum*. | |  |  |  |  |
