# Supplementary material for: Transcriptomic and proteomic analyses of a pale-green durum wheat mutant shows variations in photosystem components and metabolic deficiencies under drought stress
Source: BMC Genomics. 2014 Feb 12;15:125. doi: 10.1186/1471-2164-15-125 (PMC3937041; doi:10.1186/1471-2164-15-125)
Supplement: Additional file 4: Table S3 — Significant functional categories represented in the induced and repressed probe sets in the mutant plants under water stress conditions at anthesis. [file 1471-2164-15-125-S4.doc]

**Additional file table 3.** Significant functional categories represented in the induced and repressed probe sets in the mutant plants under water stress conditions at anthesis according to the MIPS Functional Catalogue Database (Ruepp et al., 2004). Only functional categories with a cut-off of *P* ≤ 0.005 were considered.

| **Induced** | Number of genes | *P*-value |
| --- | --- | --- |
| 20.03.01 channel / pore class transport | 2 | 2.14E-03 |
| **Repressed** |  |  |
| 01 METABOLISM | 29 | 1.79E-05 |
| 01.05 C-compound and carbohydrate metabolism | 16 | 7.08E-06 |
| 01.05.02 sugar, glucoside, polyol and carboxylate metabolism | 10 | 1.01E-04 |
| 01.20.35.01 metabolism of phenylpropanoids | 3 | 3.99E-03 |
| 02.04 glyoxylate cycle | 2 | 3.22E-04 |
| 32.01 stress response | 9 | 4.13E-04 |
| 32.01.01 oxidative stress response | 5 | 2.15E-04 |
| 32.07.07.05 peroxidase reaction | 4 | 1.26E-04 |
| 70.10.03 chromosome | 4 | 5.94E-04 |
