# Supplementary material for: Transcriptomic and proteomic analyses of a pale-green durum wheat mutant shows variations in photosystem components and metabolic deficiencies under drought stress
Source: BMC Genomics. 2014 Feb 12;15:125. doi: 10.1186/1471-2164-15-125 (PMC3937041; doi:10.1186/1471-2164-15-125)

**Additional file figure 2.** 2D-gel electrophoresis of leaf proteins from wild-type and mutant plants under water stress. Arrows represent the differentially expressed identified proteins, according to additional file table 4 and 5.

WT under water stress


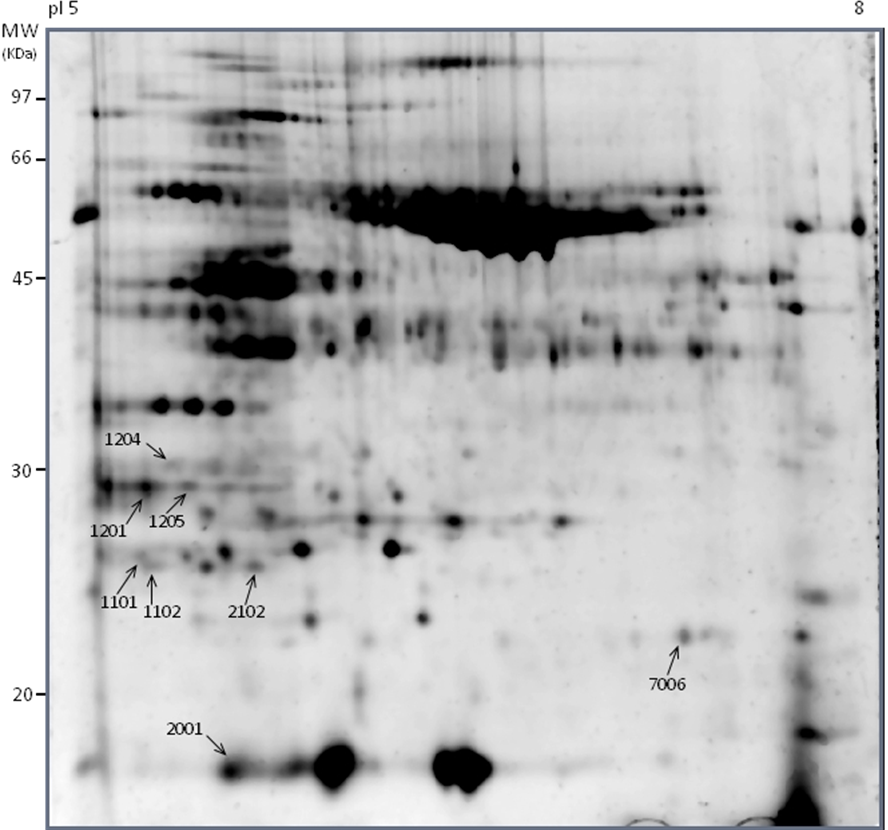


M under water stress


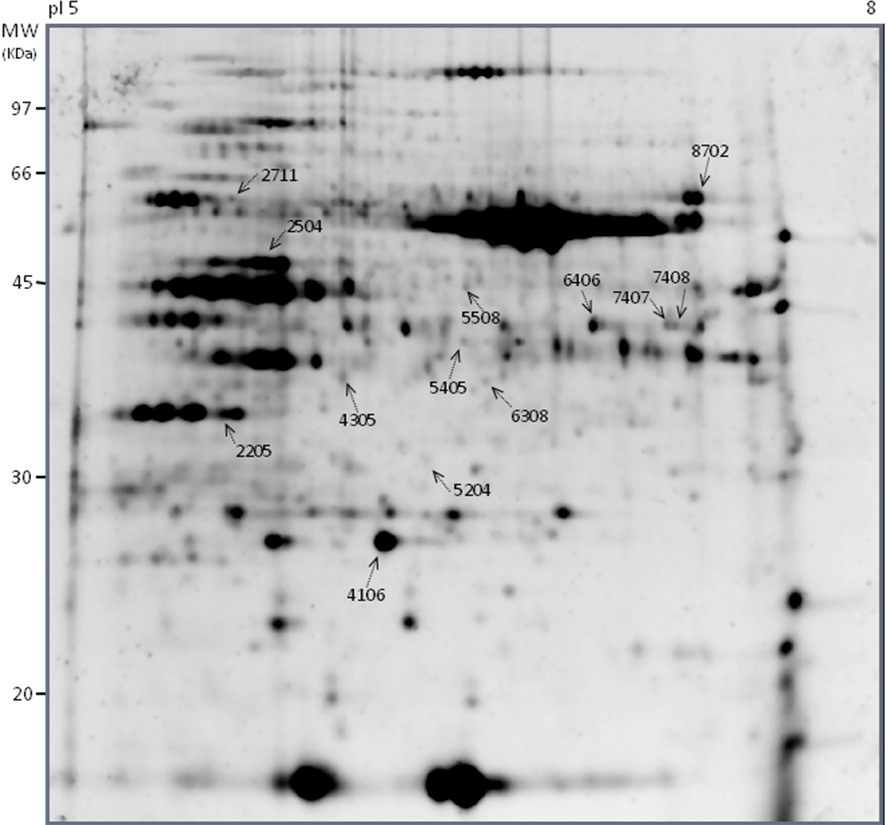

Supplement: Additional file 5: Figure S2 — 2D-gel electrophoresis of leaf proteins from wild-type and mutant plants under water stress. [file 1471-2164-15-125-S5.doc]
