# Supplementary material for: Transcriptomic and proteomic analyses of a pale-green durum wheat mutant shows variations in photosystem components and metabolic deficiencies under drought stress
Source: BMC Genomics. 2014 Feb 12;15:125. doi: 10.1186/1471-2164-15-125 (PMC3937041; doi:10.1186/1471-2164-15-125)
Supplement: Additional file 6: Table S4 — Protein identification in differently expressed spots by peptide mass fingerprinting (PMF). [file 1471-2164-15-125-S6.doc]

**Additional file table 4.** Protein identification in differently expressed spots by peptide mass fingerprinting (PMF).

| ***PMF*** |  |  |  |  |  |  |  |  |  |  |
| --- | --- | --- | --- | --- | --- | --- | --- | --- | --- | --- |
| Spot number | Protein description | SwissProt Accession number | Species^&^ | MOWSE score | % Coverage | Peptides matched | MW Thr. (KDa) | pI thr. | Start - End | Sequence |
| 7408 | G3PX; Glyceraldehyde-3-phosphate dehydrogenase, cytosolic | P26517 | 1 | 60 | 17% | 5 | 36.6 | 6.67 | 6 - 13 | IGINGFGR |
|  |  |  |  |  |  |  |  |  | 230 - 236 | LTGMSFR Oxidation (M) |
|  |  |  |  |  |  |  |  |  | 237 - 250 | VPTVDVSVVDLTVR |
|  |  |  |  |  |  |  |  |  | 274 - 293 | GIMGYVEEDLVSTDFVGDSR Oxidation (M) |
|  |  |  |  |  |  |  |  |  | 301 - 311 | AGIALNDHFVK |
| 7407 | G3PX; Glyceraldehyde-3-phosphate dehydrogenase, cytosolic | P26517 | 1 | 66 | 19% | 6 | 36.6 | 6.67 | 6 - 13 | IGINGFGR |
|  |  |  |  |  |  |  |  |  | 230 - 236 | LTGMSFR Oxidation (M) |
|  |  |  |  |  |  |  |  |  | 237 - 250 | VPTVDVSVVDLTVR |
|  |  |  |  |  |  |  |  |  | 274 - 293 | GIMGYVEEDLVSTDFVGDSR Oxidation (M) |
|  |  |  |  |  |  |  |  |  | 294 - 300 | SSIFDAK |
|  |  |  |  |  |  |  |  |  | 301 - 311 | AGIALNDHFVK |
| 2711 | ATPB; ATP synthase subunit beta, chloroplastic | P20858 | 2 | 105 | 40% | 15 | 53.9 | 5.06 | 53 - 73 | DTDDKQINVTCEVQQLLGNNR |
|  |  |  |  |  |  |  |  |  | 74 - 87 | VRAVAMSATDGLMR Oxidation (M) |
|  |  |  |  |  |  |  |  |  | 88 - 109 | GMEVIDTGAPLSVPVGGATLGR Oxidation (M) |
|  |  |  |  |  |  |  |  |  | 155 - 164 | VVDLLAPYRR |
|  |  |  |  |  |  |  |  |  | 192 - 205 | AHGGVSVFGGVGER |
|  |  |  |  |  |  |  |  |  | 208 - 217 | EGNDLYMEMK |
|  |  |  |  |  |  |  |  |  | 232 - 246 | VALVYGQMNEPPGAR Oxidation (M) |
|  |  |  |  |  |  |  |  |  | 249 - 261 | VGLTALTMAEYFR |
|  |  |  |  |  |  |  |  |  | 249 - 261 | VGLTALTMAEYFR Oxidation (M) |
|  |  |  |  |  |  |  |  |  | 266 - 277 | QDVLLFIDNIFR |
|  |  |  |  |  |  |  |  |  | 278 - 291 | FVQAGSEVSALLGR |
|  |  |  |  |  |  |  |  |  | 292 - 312 | MPSAVGYQPTLSTEMGSLQER |
|  |  |  |  |  |  |  |  |  | 360 - 378 | GIYPAVDPLDSTSTMLQPR Oxidation (M) |
|  |  |  |  |  |  |  |  |  | 379 - 390 | IVGNEHYETAQR |
|  |  |  |  |  |  |  |  |  | 391 - 397 | VKETLQR |
| 2205 | PSBO; Oxygen-evolving enhancer protein 1, chloroplastic (PSII) | Q40459 | 3 | 79 | 27% | 7 | 35.4 | 5.89 | 37 - 46 | AFGVEPAAAR |
|  |  |  |  |  |  |  |  |  | 91 - 100 | RLTFDEIQSK |
|  |  |  |  |  |  |  |  |  | 136 - 146 | FCLEPTSFTVK |
|  |  |  |  |  |  |  |  |  | 167 - 187 | LTYTLDEIEGPFEVSSDGTVK |
|  |  |  |  |  |  |  |  |  | 188 - 208 | FEEKDGIDYAAVTVQLPGGER |
|  |  |  |  |  |  |  |  |  | 192 - 208 | DGIDYAAVTVQLPGGER |
|  |  |  |  |  |  |  |  |  | 248 - 264 | GGSTGYDNAVALPAGGR |
| 4106 | PSBP; Oxygen-evolving enhancer protein 2, chloroplastic (PSII) | Q00434 | 2 | 142 | 47% | 11 | 27.4 | 8.84 | 87 - 100 | KNTDFVAYSGEGFK |
|  |  |  |  |  |  |  |  |  | 88 - 100 | NTDFVAYSGEGFK |
|  |  |  |  |  |  |  |  |  | 107 - 113 | WNPSKER |
|  |  |  |  |  |  |  |  |  | 112 - 121 | EREFPGQVLR |
|  |  |  |  |  |  |  |  |  | 114 - 121 | EFPGQVLR |
|  |  |  |  |  |  |  |  |  | 122 - 141 | YEDNFDATSNLSVIINPTTK |
|  |  |  |  |  |  |  |  |  | 122 - 142 | YEDNFDATSNLSVIINPTTKK |
|  |  |  |  |  |  |  |  |  | 170 - 197 | TDSEGGFESDAVATANVLESSAPVVDGK |
|  |  |  |  |  |  |  |  |  | 198 - 207 | QYYSITVLTR |
|  |  |  |  |  |  |  |  |  | 208 - 228 | TADGDEGGKHQLITATVADGK |
|  |  |  |  |  |  |  |  |  | 246 - 258 | KFVENAAGSFSVA |
| 2001 | RuBisCoS2; Ribulose bisphosphate carboxylase small chain PW9, chloroplastic | P26667 | 2 | 92 | 42% | 7 | 19.8 | 8.52 | 58 - 75 | KFETLSYLPPLSTEALLK |
|  |  |  |  |  |  |  |  |  | 76 - 82 | QVDYLIR |
|  |  |  |  |  |  |  |  |  | 100 - 111 | EHNSSPGYYDGR |
|  |  |  |  |  |  |  |  |  | 139 - 146 | EYPDAYVR |
|  |  |  |  |  |  |  |  |  | 147 - 154 | VIGFDNMR Oxidation (M) |
|  |  |  |  |  |  |  |  |  | 155 - 174 | QVQCVSFIAFRPPGCEESGK |
|  |  |  |  |  |  |  |  |  | 155 - 175 | QVQCVSFIAFRPPGCEESGKA |
| 6308 | Isoflavone reductase | B5M699* | 1 | 73 | 21% | 6 | 36.1 | 6.16 | 12 - 23 | VLVIGGTGYIGR |
|  |  |  |  |  |  |  |  |  | 24 - 31 | FIVAASAR |
|  |  |  |  |  |  |  |  |  | 32 - 41 | EGHPTAVLVR |
|  |  |  |  |  |  |  |  |  | 51 - 58 | AAVLQGFR |
|  |  |  |  |  |  |  |  |  | 113 - 124 | FFPSEYGNDVDR |
|  |  |  |  |  |  |  |  |  | 144 - 163 | AIEAEGIPYTYVSSNFFAGR |
| 1101 | HBL2; Non-symbiotic hemoglobin 2 | O24521 | 4 | 73 | 48% | 6 | 17.9 | 5.4 | 2 - 9 | GEIGFTEK |
|  |  |  |  |  |  |  |  |  | 16 - 27 | ESWEILKQDIPK |
|  |  |  |  |  |  |  |  |  | 45 - 62 | GLFSFLRDSDEVPHNNPK |
|  |  |  |  |  |  |  |  |  | 65 - 72 | AHAVKVFK |
|  |  |  |  |  |  |  |  |  | 83 - 103 | EEGKVVVADTTLQYLGSIHLK |
|  |  |  |  |  |  |  |  |  | 121 - 129 | TLKEGLGEK |
| 1205 | C71AJ; Cytochrome P450 71A19 | Q9T0K0 | 4 | 74 | 22% | 8 | 55.9 | 8.92 | 1 - 20 | MEIILVTLCLTTLLALLLLK |
|  |  |  |  |  |  |  |  |  | 25 - 39 | RTTTNNLNLPPSPWR |
|  |  |  |  |  |  |  |  |  | 105 - 111 | VIDKILR |
|  |  |  |  |  |  |  |  |  | 115 - 129 | DVAFAPYGEYWKQMK |
|  |  |  |  |  |  |  |  |  | 150 - 159 | EDEIKLMIEK |
|  |  |  |  |  |  |  |  |  | 365 - 373 | VFSEDVTLK |
|  |  |  |  |  |  |  |  |  | 432 - 453 | ICPGIGFTSALIGVTLANIVKR |
|  |  |  |  |  |  |  |  |  | 481 - 490 | FPLIAIPSSA |
| 8702 | CATA1; Catalase-1 | Q43206 | 2 | 158 | 45% | 19 | 57.0 | 6.52 | 38 - 50 | GPILLEDYHLVEK |
|  |  |  |  |  |  |  |  |  | 51 - 58 | IADFDRER |
|  |  |  |  |  |  |  |  |  | 73 - 91 | GFFEVTHDVSHLTCADFLR |
|  |  |  |  |  |  |  |  |  | 92 - 102 | APGVQTPVIVR |
|  |  |  |  |  |  |  |  |  | 103 - 110 | FSTVIHER |
|  |  |  |  |  |  |  |  |  | 111 - 120 | GSPETLRDPR |
|  |  |  |  |  |  |  |  |  | 130 - 146 | EGNWDLVGNNFPVFFIR |
|  |  |  |  |  |  |  |  |  | 147 - 163 | DGMKFPDMVHALKPNPK 2 Oxidation (M) |
|  |  |  |  |  |  |  |  |  | 164 - 171 | THIQENWR |
|  |  |  |  |  |  |  |  |  | 201 - 215 | HMDGSGVNTYTLVNR Oxidation (M) |
|  |  |  |  |  |  |  |  |  | 234 - 253 | SLLEEEAVTVGGTNHSHATK |
|  |  |  |  |  |  |  |  |  | 292 - 305 | TWPEDVVPLQPVGR |
|  |  |  |  |  |  |  |  |  | 345 - 353 | IFSYSDTQR |
|  |  |  |  |  |  |  |  |  | 356 - 369 | LGPNYLLLPANAPK |
|  |  |  |  |  |  |  |  |  | 387 - 396 | DEEVDYFPSR |
|  |  |  |  |  |  |  |  |  | 425 - 434 | ENNFKQPGER |
|  |  |  |  |  |  |  |  |  | 435 - 442 | YRSMDPAR Oxidation (M) |
|  |  |  |  |  |  |  |  |  | 437 - 445 | SMDPARQER Oxidation (M) |
|  |  |  |  |  |  |  |  |  | 450 - 458 | WIDALSDPR |

* Accession number from Trembl.

^&^Species: 1: *Hordeum vulgare*; 2: *Triticum aestivum*; 3: *Nicotiana tabacum*; 4: *Arabidopsis thaliana*.
