# Supplementary material for: Transcriptomic and proteomic analyses of a pale-green durum wheat mutant shows variations in photosystem components and metabolic deficiencies under drought stress
Source: BMC Genomics. 2014 Feb 12;15:125. doi: 10.1186/1471-2164-15-125 (PMC3937041; doi:10.1186/1471-2164-15-125)
Supplement: Additional file 7: Table S5 — Protein identification in differently expressed spots by MS/MS analysis. [file 1471-2164-15-125-S7.doc]

**Additional file table 5.** Protein identification in differently expressed spots by MS/MS analysis.

| ***MS/MS*** |  |  |  |  |  |  |  |  |  |  |  |
| --- | --- | --- | --- | --- | --- | --- | --- | --- | --- | --- | --- |
| Spot number | Protein description | SwissProt Accession number | Species^&^ | Ion score | % Coverage | Peptides matched | MW Thr. (KDa) | pI Thr. | Start - End | Sequence | Partial Ion score |
| 1102 | Chlorophyll a-b binding protein 1B-21, chloroplastic (CAB 1B-21) | Q9SDM1 | 1 | 36 | 4% | 1 | 26.7 | 5.84 | 83 - 92 | FKESEIYHCR |  |
| 1201 | Chloroplast light-harvesting chlorophyll a/b binding protein (Lhc II typeI CAB) | ADL41158* | 3 | 44 | 3% | 1 | 28.4 | 5.14 | 95 - 104 | NRELEVIHCR |  |
| 1204 | Precursor of CP29, core chlorophyll a/b binding (CAB) protein (PSII Lhc4) | CAA44777* | 1 | 41 | 3% | 1 | 30.8 | 5.33 | 116 - 125 | YQAFELIHAR |  |
| 2102 | Chlorophyll a-b binding protein CAB 1B-21 (PSI Lhc I) | ACO06083* | 3 | 40 | 3% | 1 | 27.2 | 5.42 | 85 - 94 | FKESEIYHCR |  |
| 7006 | Photosystem I reaction center subunit IV, chloroplastic (PSI RC sub IV) | P13194 | 1 | 26 | 4% | 1 | 15.4 | 9.82 | 118 - 123 | YPVVVR |  |
| 2504 | Elongation factor Tu, chloroplastic-like, predicted | XP_003575279* | 4 | 281 | 11% | 4 | 50.6 | 5.88 | 108 - 119 | KYDEIDAAPEER | 96 |
|  |  |  |  |  |  |  |  |  | 122 - 137 | GITINTATVEYETETR | 45 |
|  |  |  |  |  |  |  |  |  | 201 - 217 | DQVDDEELLELVDLEVR | 67 |
|  |  |  |  |  |  |  |  |  | 311 - 321 | VGDPVDLVGIR | 73 |
| 4305 | Cysteine synthase | P38076 | 3 | 37 | 3% | 1 | 34.2 | 5.48 | 284 - 293 | VAQRPENAGK |  |
| 5204 | Putative 3-beta hydroxysteroid dehydrogenase/isomerase protein | BAJ86066* | 1 | 67 | 6% | 2 | 35.0 | 8.92 | 250 - 261 | AGGLQDKDGGVR | 36 |
|  |  |  |  |  |  |  |  |  | 320 - 328 | SVFAQIATR | 31 |
| 5405 | Glyceraldehyde-3-phosphate dehydrogenase A, chloroplastic | P09315 | 2 | 41 | 2% | 1 | 43.2 | 7 | 320 - 330 | TLAEEVNQAFR |  |
| 5508 | Chloroplast aspartate aminotransferase | ACG59771* | 3 | 145 | 10% | 4 | 41.0 | 6.69 | 2 - 10 | LNLGVGAYR | 23 |
|  |  |  |  |  |  |  |  |  | 68 - 81 | VATLQSLSGTGSLR | 66 |
|  |  |  |  |  |  |  |  |  | 82 - 89 | LAAAFIQR | 24 |
|  |  |  |  |  |  |  |  |  | 221 - 228 | NLGLYAER | 32 |
| 6406 | Fructose-bisphosphate aldolaseF2CR16 | BAJ85287* | 1 | 194 | 8% | 3 | 38.1 | 6.06 | 239 - 253 | VGAEVIAEYTVAALR | 51 |
|  |  |  |  |  |  |  |  |  | 239 - 254 | VGAEVIAEYTVAALRR | 78 |
|  |  |  |  |  |  |  |  |  | 314 - 326 | ENVADAQATFLAR | 65 |

*Accession number from NCBI.

^&^Species: 1: *Hordeum vulgare*; 2: *Zea mays*; 3: *Triticum aestivum*; 4: *Brachypodium distachyon*.
