# Supplementary figures and images for: Transcriptomic and proteomic analyses of a pale-green durum wheat mutant shows variations in photosystem components and metabolic deficiencies under drought stress
Source: BMC Genomics. 2014 Feb 12;15:125. doi: 10.1186/1471-2164-15-125 (PMC3937041; doi:10.1186/1471-2164-15-125)

**Additional file figure 3.** Rain and irrigation pattern in the field experiments.


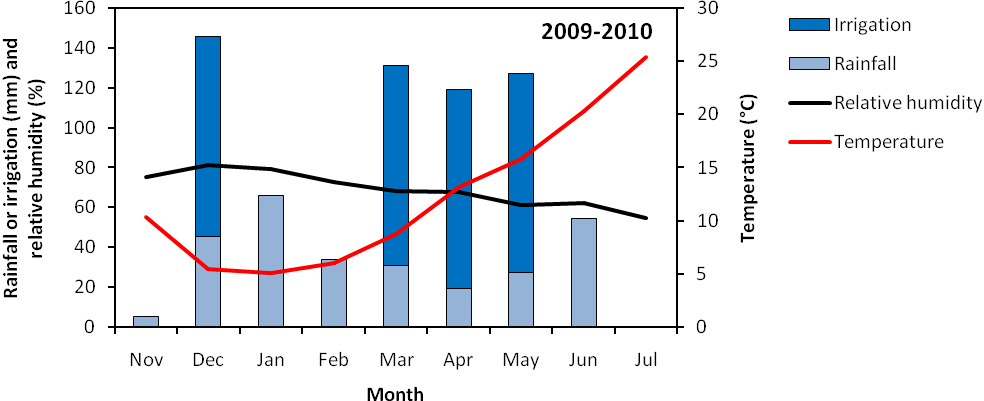


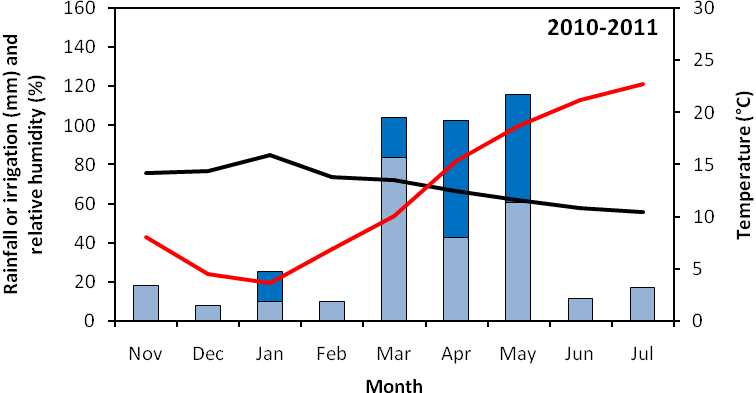


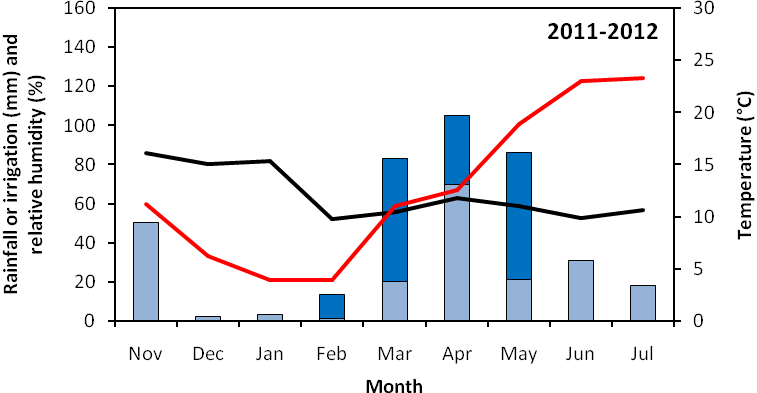

Supplement: Additional file 8: Figure S3 — Rain and irrigation pattern in the field experiments. [file 1471-2164-15-125-S8.doc]
