# Supplementary material for: Transcriptomic and proteomic analyses of a pale-green durum wheat mutant shows variations in photosystem components and metabolic deficiencies under drought stress
Source: BMC Genomics. 2014 Feb 12;15:125. doi: 10.1186/1471-2164-15-125 (PMC3937041; doi:10.1186/1471-2164-15-125)
Supplement: Additional file 9: Table S6 — Sequence of primers used for qRT-PCR. [file 1471-2164-15-125-S9.doc]

**Additional file table 6.** Sequence of primers used for qRT-PCR.

| **Probe set** | **Gene** | **NCBI ID** | **Primer** | **Sequence 5’-3’** | **Tm** | **Length (bp)** |
| --- | --- | --- | --- | --- | --- | --- |
| Ta.28253.1.S1_at | Actin | GQ339780 | Forward | GTTCCGTTGCCCTGAGGTCCTT | 60 | 148 |
|  |  |  | Reverse | TGAGCCACCACTGAGCACAATGTTA | 60 |  |
| TaAffx.128418.43.S1_at | Chitinase 3 | AB029936 | Forward | GCTACTGTGACCTCCTCGGTGTCA | 59 | 150 |
|  |  |  | Reverse | GGTCTATCGCCACGCCATTGTTAC | 60 |  |
| TaAffx.6092.1.S1_at | Naringenin,2-oxoglutarate 3-dioxygenase | BJ320006* | Forward | CACCTTCAGCGACTACCGCAAGTAC | 60 | 200 |
|  |  |  | reverse | TCCCAACTGCTGGCAACCTATAACA | 61 |  |
| TaAffx.26815.1.S1_at | Blufensin 1 | AK333112* | Forward | CCGAGGATGGCAAAGAACAACTC | 59 | 188 |
|  |  |  | Reverse | GGAAATGGTTCATCTCGTGCTTATG | 58 |  |
| Ta.169.1.S1_x_at | Germin-like protein (glp2b) | AJ237943 | Forward | GGTCAGACCCACCAATATCAGATGA | 58 | 165 |
|  |  |  | Reverse | AACTCAACCGTGTCCTTCACTTGTG | 58 |  |
| Ta.8614.2.S1_x_at | WRKY45-like transcription factor | AB603889 | Forward | ATGGCGCAAGTACGGGCAGA | 60 | 159 |
|  |  |  | Reverse | TGCACGCCGATGTAGGTGACC | 60 |  |
| Ta.8228.1.S1_at | Agmatine coumaroyltransferase-1 | AY234333* | Forward | CTGCTCATCCTGCTACCGTCCTTCT | 61 | 120 |
|  |  |  | Reverse | CAAGCGGAGCTAGTCGAGGGTGTAG | 61 |  |
| Ta.2638.1.S1_at | RAB | X62476 | Forward | GGCATCATGGACAAGATCAAGGA | 58 | 169 |
|  |  |  | Reverse | ACTAGGCTCACGTTGTATGGTGGAA | 58 |  |
| Ta.13255.1.S1_at | Dehydrin WZY1 | AF453444 | Forward | ATGGCACCTACGGACAGCAGGGTA | 62 | 128 |
|  |  |  | Reverse | ATCGGTGACATGCGTCCCAGTAC | 60 |  |
| TaAffx.98394.1.S1_at | Q-type C2H2 zinc fingerprotein (ZFP23) | EU408223 | Forward | GAGCCAGGGCTTCGACCTGAAT | 60 | 119 |
|  |  |  | Reverse | GCATGAGCCTCGGCTTCTTGAA | 60 |  |
